# Hevin Promotes Aging‐Related Cardiac Dysfunction via Facilitating Cardiac Inflammation in Male Mice

**DOI:** 10.1111/acel.70369

**Published:** 2026-01-11

**Authors:** Shi‐Yu Huang, Yu‐Jie Chen, Yu‐Xin Hu, Jia‐Chen Liu, Min Hu

**Affiliations:** ^1^ Department of Ultrasound Sun Yat‐Sen University Cancer Center, State Key Laboratory of Oncology in South China, Collaborative Innovation Center for Cancer Medicine, Guangdong Provincial Clinical Research Center for Cancer Guangzhou Guangdong China; ^2^ Department of Urology Renmin Hospital of Wuhan University Wuhan Hubei China; ^3^ McGill University Montréal Québec Canada; ^4^ Department of Cardiology Renmin Hospital of Wuhan University Wuhan Hubei China

**Keywords:** CCL5, Hevin, inflammation, TLR4

## Abstract

As individuals age, there is a gradual increase in the levels of inflammation in the body, with macrophages, essential immune cell types, assuming a critical role in modulating inflammatory responses and eliminating senescent cells. Prolonged inflammatory reactions can result in tissue damage, the advancement of diseases, and the acceleration of aging processes. Hevin (also known as SPARCL1, secreted protein acidic and rich in cysteine‐like protein 1) is involved in regulating inflammatory responses and the polarization of macrophages. The current study seeks to elucidate the role of Hevin in the context of cardiac aging. Aging or young C57 BL/6 male mice were intravenously injected with Hevin or knocked down Hevin with adeno‐associated virus serotype 9 (AAV9) vectors. To screen the underlying mechanisms, RNA‐seq was used. Meanwhile, RAW264.7 cells were employed to investigate the role of Hevin in macrophage polarization. Aging mice displayed elevated Hevin serum levels compared to their younger counterparts, along with increased Hevin expression associated with poor cardiac function. Administration of Hevin enhanced aging‐related cardiac remodeling, whereas Hevin knockout ameliorated such remodeling and dysfunction. RNA‐seq analysis unveiled that Hevin triggered CCL5 activation in aging hearts, and blocking CCL5 reversed the adverse effects of Hevin‐induced cardiac aging in vivo. Functionally, circulating Hevin released by iWAT stimulated cardiac macrophages via TLR4, prompting their polarization and CCL5 release, exacerbating cardiac dysfunction and attracting more inflammatory cells for the secretion of pro‐inflammatory factors. During aging, Hevin expression inversely correlates with cardiac function, and its absence effectively mitigates aging‐related cardiac dysfunction by diminishing inflammatory responses. Our study uniquely identifies Hevin as a promising predictive and therapeutic target for cardiac aging.

## Graphical abstract

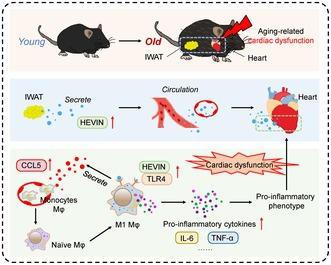

## 1 Introduction

Aging significantly increases the likelihood of developing cardiovascular diseases, with age‐related heart issues playing a crucial role in the health outcomes of the elderly, impacting both morbidity and mortality rates (Triposkiadis et al. 2019). As we age, multiple processes likely contribute to cardiac dysfunction, including fibrosis, inflammation, mechanical stiffening, and diastolic dysfunction, and a growing imbalance between loss and birth of cardiomyocyte. These processes are driven by molecular mechanisms such as telomere shortening, senescence‐associated secreted factors, accumulation of somatic mutations, epigenetic changes, and alterations in noncoding RNAs regulating gene expression (Chen et al. 2022; Li, Hastings, et al. 2020). Even though the precise molecular and cellular foundations remain incompletely comprehended, chronic cardiac inflammation has been associated with the development of aging‐related cardiac dysfunction in an inflammaging‐dependent fashion (Liberale et al. 2022). In elderly individuals, the presence of heightened levels of various inflammatory cytokines such as interleukin‐6 (IL‐6), IL‐8, IL‐1β, and tumor necrosis factor‐α (TNF‐α) in both serum and cardiac tissue is closely linked to their cardiac functional metrics and long‐term outlook. Furthermore, dampening inflammation has demonstrated cardiac advantages for specific individuals (Gottdiener et al. 2000; Hu et al. 2022). Aguilar and coworkers found that suppression of NLRP3 prevented many age‐associated changes in the heart, preserved cardiac function of aged mice, and increased lifespan (Marin‐Aguilar et al. 2020). Indeed, a state of chronic sterile low‐grade inflammation characterizes older organisms (also known as inflamm‐aging). Mediators of inflamm‐aging, such as TNF‐α and IL‐1β can contribute to age‐related progression of cardiac‐dysfunction (De Miguel et al. 2015; Liberale et al. 2020). Hence, targeting inflammation presents a logical approach for addressing cardiac dysfunction associated with aging.

Inflamm‐aging includes chronic activation of the innate immune system and increased circulating levels of pro‐inflammatory mediators, such as IL‐1β, IL‐6, TNF‐α, and of the biomarker C‐reactive protein (CRP) (Gerli et al. 2000; Puzianowska‐Kuznicka et al. 2016). Inflamm‐aging and metaflammation show similar molecular mechanisms with sterile inflammatory response as a critical determinant and macrophages as master cells (Franceschi et al. 2018; Nardini et al. 2018; Prattichizzo et al. 2018). Macrophages are one of the most active cell types participating in the cardiac remodeling events that occur during the inflammatory (Yap et al. 2023; Zaman and Epelman 2022). Aging causes a shift in macrophage polarization from anti‐inflammatory ‘M2’ to proinflammatory ‘M1’ that is associated with a rise in cytokines (Becker et al. 2018) M1‐like macrophages are defined as macrophages that produce pro‐inflammatory cytokines and mediate resistance to intracellular pathogens, but these also lead to tissue destruction. M2‐like macrophages are, in turn, involved in anti‐inflammatory responses and tissue repair/remodeling (Nicolas‐Avila et al. 2022). C‐C chemokine motif ligand 5 (CCL5), also called RANTES, is generated by a variety of cell types like platelets, macrophages, and eosinophils. It acts as a potent chemoattractant, guiding leukocytes to inflammatory sites in different disease conditions (Marques et al. 2013; Marra and Tacke 2014). Especially, CCL5 can directly activate M1‐like polarization and impede M2‐like polarization (Li, Sun, et al. 2020). It has been reported that CCL5 exerts its biological effects by engaging C‐C chemokine motif receptor 1 (CCR1), CCR3, and CCR5 receptors on cell surfaces, activating signaling pathways like the signal transducer and activator of transcription factor 3, nuclear factor (NF)‐κb, and mitogen‐activated protein kinase (MAPK), observed in conditions like non‐alcoholic fatty liver disease (NAFLD), atherosclerosis, and myocardial infarction (Li, Sun, et al. 2020; Montecucco et al. 2012).

Hevin is recognized as a matricellular protein with multifaceted roles across diverse disease states (Gagliardi et al. 2017). Numerous studies have demonstrated that Hevin plays a pivotal role in various diseases by modulating inflammatory responses. A prior investigation revealed elevated plasma levels of Hevin in non‐alcoholic steatohepatitis (NASH), showing a correlation with the disease's severity in a human cohort (Liu et al. 2021). Significantly, Vaughan et al. discovered that Hevin activates macrophages through Toll‐like receptor 4 (TLR4), inducing a proinflammatory response in pneumonia (Zhao et al. 2024). Likewise, the interaction between Hevin and TLR4 triggers the activation of the NF‐κB/p65 signaling pathway, fostering the upregulation of C‐C motif chemokine ligand 2 (CCL2) expression in hepatocytes (Liu et al. 2021). Zhang and colleagues elucidated that Hevin facilitates macrophage recruitment by activating WNT/β‐catenin signaling, thereby inducing the secretion of chemokine ligand 5 in osteosarcoma cells (Zhao et al. 2018). Previous studies have found that secreted proteins such as Fibronectin type III domain‐containing5 and Isthmin‐1 can influence age‐related cardiac dysfunction by modulating inflammatory responses (Hu et al. 2022; Hu, Zhang, Gao, et al. 2024). As of now, there exists a dearth of research concerning the involvement and mechanisms of Hevin in cardiac function. In the current investigation, our objective is to elucidate the role of Hevin in age‐related cardiac dysfunction and delve into the associated mechanisms.

## 2 Materials and Methods

### 2.1 Reagents

Senescence‐Associated β‐Galactosidase (SA‐β gal) Staining Kit (#9860) was purchased from Cell Signaling Technology (Danvers, MA, USA). 3‐Nitrotyrosine (3‐NT) ELISA Assay Kit (#ab116691) was purchased from Abcam (Cambridge, UK). AAV9 vectors carrying the short hairpin RNA against Hevin (sh*Hevin*) or scrambled sh*RNA* were synthesized by Vigene Bioscience. Interleukin‐6 (IL‐6) Mouse ELISA Kit (#KMC0061), Tumor Necrosis Factor‐α (TNF‐α) Mouse ELISA Kit (#BMS607‐3), Alexa Fluor 488 conjugated Wheat Germ Agglutinin (WGA, #W11261), and SlowFade Gold Antifade Reagent with DAPI (#S36939) were purchased from Invitrogen (Carlsbad, CA, USA). Mouse N‐Terminal Pro‐Brain Natriuretic Peptide (NT‐proBNP) ELISA Kit (#MBS2124067) and Mouse Hevin ELISA Kit (#MBS2533471) were purchased from MyBioSource Inc. (San Diego, CA, USA). Recombinant mouse Hevin (#4547‐SL) was purchased from R&D system (Minneapolis, Minnesota, USA). UK‐427857 was purchased from GlaxoSmithKline (London, UK). 4‐Hydroxynonenal (4‐HNE) ELISA Kit (#H268) was purchased from Nanjing Jiancheng Bioengineering Institute (Nanjing, China).

### 2.2 Animals and Treatments

Male C57BL/6 mice were accommodated in a specific pathogen‐free barrier facility and given ad libitum access to a standard laboratory chow diet. After 1 week acclimation, 8‐month (M)‐old young and 20‐M‐old aging mice received a single intravenous injection of AAV‐sh*Hevin* at a dose of 1 × 10^11^ viral genome to knock down Hevin and sacrificed after 2 months Hu, Zhang, Gao, et al. (2024). For Hevin treatment, recombinant mouse Hevin protein (0.2 mg/kg) was intraperitoneally injected into mice. To inhibit CCR5, aging mice were treatment with UK‐427857 (80 mg/kg/day) (Ishihara et al. 2022). In the neutralizing study, the mice were intraperitoneally injected with a neutralizing antibody (NAb) against CCL5 (0.1 mg/mouse) or isotype‐controlled IgG (Montecucco et al. 2012). All animal procedures were authorized by the Animal Care and Use Committee of Renmin Hospital, Wuhan University, and were conducted following the Guidelines for Care and Use of Laboratory Animals issued by the US National Institutes of Health (Publication No. 85–23, revised 1996).

### 2.3 Human Serum Samples

Serum samples from aged (> 60 years) and age‐matched young (< 60 years) patients were collected with informed consent from the patients and their families to measure circulating Hevin levels. The measured serum Hevin levels were then subjected to Pearson correlation analysis with clinical parameters such as NT‐proBNP and ejection fraction recorded in the patients' medical records. All experimental procedures involving human samples in this study were in accordance with the Declaration of Helsinki and written informed consent was obtained from all donors or their legal guardians, and all experimental procedures involving human samples in this study were approved by the Review Board of Renmin Hospital of Wuhan University.

### 2.4 Echocardiography

Conscious mice underwent echocardiography using a Vevo 3100 high‐resolution Preclinical Imaging System (FUJIFILM Visual Sonics, Toronto, Canada) equipped with a 30‐MHz MX 400 linear ultrasound transducer as previously outlined. The procedure involved standard 2D‐guided M‐mode echocardiography to measure left ventricle internal diameters at diastole (LVIDd) and systole (LVIDs). Fractional shortening (FS) was determined using the formula: (LVIDd‐LVIDs)/LVIDd×100%. Tissue Doppler imaging was utilized to assess diastolic function by calculating the E/A ratio, representing the early (E) to late (A) ventricular filling velocities.

### 2.5 Histology

Cardiomyocyte cross‐sectional area was assessed through WGA staining. Initially, murine hearts were excised, fixed in a 10% formalin solution for 48 h, and then dehydrated, embedded in paraffin, and sliced into 5 μm sections. Subsequently, these heart slices were subjected to WGA staining by immersing them in WGA working buffer (1:200) at 37°C for 1 h. A minimum of 200 cells per group were examined, and Image‐Pro Plus 6.0 software was utilized to analyze the cardiomyocyte cross‐sectional areas. For collagen evaluation, heart sections underwent picrosirius red (PSR) staining, with at least 60 fields per group included in a blinded manner for interstitial fibrosis analysis.

### 2.6 SA‐β Gal Staining

To assess cellular senescence, SA‐β gal staining was conducted on heart slices and cell coverslips following established protocols (Liang et al. 2020; Sun et al. 2021). Initially, fresh frozen heart slices and cell coverslips were fixed with a fixation buffer at room temperature for 15 min. Subsequently, they were treated with the staining solution at 37°C for 24 h. The percentage of SA‐β gal+ cells was determined blindly under a light microscope, with a minimum of 5 high‐magnification fields analyzed per mouse.

### 2.7 Immunofluorescence Staining

Immunofluorescence staining involved deparaffinization and hydration of heart slices, followed by high‐pressure antigen retrieval in citrate (pH = 6.0). For in vitro immunofluorescence staining, cell coverslips underwent fixation in 4% formaldehyde and permeabilization in 1% Triton X‐100. Subsequently, heart slices or cell coverslips were incubated with 10% goat serum to inhibit non‐specific reactions, and then exposed to primary antibodies (refer to Table S1) overnight at 4°C, followed by incubation with Alexa Fluor secondary antibodies (1:200 dilution) at 37°C for an additional hour. Cell nuclei were counterstained with SlowFade Gold antifade reagent containing DAPI, and images were captured using a DP74 fluorescence microscope (OLYMPUS, Tokyo, Japan).

### 2.8 Measurements of Telomere Length

Telomere length was determined following established procedures. Genomic DNA was extracted from the heart samples, and the telomere length was quantified by calculating the ratio of telomere repeat copy number to the copy number of a single‐gene, acidic ribosomal phosphoprotein PO forward (36B4).

### 2.9 Measurements of Lipofuscin Content

Myocardial lipofuscin was extracted and assessed following standard protocols. Fresh heart samples were homogenized in a chloroform‐methanol solution (1:20, w:v), and the lipofuscin content in the chloroform‐rich layer was quantified using an excitation/emission wavelength of 350/485 nm.

### 2.10 Adipose Tissue Transplantation

Adipose tissue was transplanted according to previous studies (Jiang et al. 2022; Wang et al. 2021). The mice were anesthetized by the intraperitoneal injection of pentobarbital sodium (50 mg/kg). To obtain BAT, the interscapular skin was incised, and the BAT was harvested from aged mice. To obtain WAT, the inguinal white adipose tissue and epididymal white adipose tissue of the aged mice were dissected and harvested. The harvested adipose tissue was collected on a sterile petri dish with saline, and the residual blood and other tissues were washed away by PBS. Then the adipose tissue was cut into pieces with sterile ophthalmic scissors for about 2 min and transferred to a sterile syringe. For the transplantation, 0.15 mL adipose tissue was injected subcutaneously in each flank with a 15‐gauge cannula.

### 2.11 Isolation and Culture of Mice Cardiac Cell Populations

Adult mouse cardiac cells were isolated as described previously (Ackers‐Johnson et al. 2016; Dick et al. 2019; Pinto et al. 2016). Briefly, mouse hearts were minced and enzymatically digested with collagenase II, dispase II, and DNase I to obtain single‐cell suspensions. Cardiomyocytes were isolated using the Langendorff perfusion method and cultured short‐term on laminin‐coated plates in M199 medium. Cardiac fibroblasts were obtained by differential adhesion and maintained in DMEM with 10% FBS. Cardiac macrophages were sorted and cultured in RPMI‐1640 containing 10% FBS and M‐CSF. Endothelial cells were isolated using CD31‐conjugated magnetic beads and grown on fibronectin‐coated plates in EGM‐2 medium.

### 2.12 NRCM Culture and Treatment

Neonatal rat cardiomyocytes (NRCMs) were isolated and maintained in DMEM/F12 supplemented with 15% fetal bovine serum (FBS), following our previously reported protocol (Hu, Zhang, Hu, et al. 2024; Ma et al. 2023). NRCM was treated with Hevin or conditioned medium (ConM). ConM was collected from these RAW264.7 cells in the presence of Hevin for 24 h.

### 2.13 RNA‐Seq

The hearts from 20‐month‐old mice receiving Hevin or Saline treatment underwent RNA‐sequencing. Total RNA was extracted and assessed for quality using the RNA 6000 Nano kit on the Bioanalyzer 2100 system (Agilent Technologies, CA, USA). RNA‐seq and data analyses were carried out by Bioyi Biotechnology Co. Ltd. (Wuhan, China).

### 2.14 Isolation and Culture of Monocyte‐Derived Mφ

Human peripheral blood monocytes were isolated as described (Berliner et al. 1990; Uzui et al. 2002; Xu et al. 1999). Briefly, peripheral blood mononuclear cells were obtained from healthy donors using Ficoll‐Paque density gradient centrifugation. Following removal of nonadherent cells, the remaining mononuclear cells were cultured in RPMI 1640 medium supplemented with 10% fetal calf serum, 24 mmol/L NaHCO₃, 25 mmol/L HEPES, 100 U/mL penicillin, 100 μg/mL streptomycin, 1 mmol/L sodium pyruvate, 4 mmol/L L‐glutamine, and nonessential amino acids. The cells were maintained in a humidified atmosphere at 37°C for 7 days.

### 2.15 Western Blot

Total proteins were extracted, separated by SDS‐PAGE, and transferred onto PVDF membranes using established techniques. The membranes were then treated with primary antibodies (refer to Table S1) overnight at 4°C, followed by incubation with HRP‐conjugated secondary antibodies at room temperature for 1 h. Visualization was carried out using an electrochemiluminescence reagent and a Bio‐Rad ChemiDoc XRS+ System.

### 2.16 Quantitative Real‐Time PCR

After total RNA extraction, cDNA synthesis was conducted using the Transcriptor First Strand cDNA Synthesis Kit (Roche, Basel, Switzerland). Subsequently, quantitative real‐time PCR was carried out on the Roche LightCycler480 system utilizing the SYBR Green IMaster Mix (Roche), with primer sets detailed in Table S2.

### 2.17 Statistical Analysis

The data were presented as mean ± standard deviation (S.D.) and analyzed using GraphPad Prism (version 8.0). Unpaired two‐tailed Student's *t*‐tests were employed to compare differences between two groups with normal distribution and variance homogeneity. For multiple comparisons, one‐way analysis of variance (ANOVA) followed by Tukey's post hoc test was conducted. Pearson's correlation coefficients were utilized to assess correlations, while the *χ* ^2^ test was applied for comparing categorical variables. Statistical significance was set at a *p*‐value below 0.05.

## 3 Results

### 3.1 Hevin Elevates in Aging Mouse Circulation and Correlates With Cardiac Function

**FIGURE 1:**
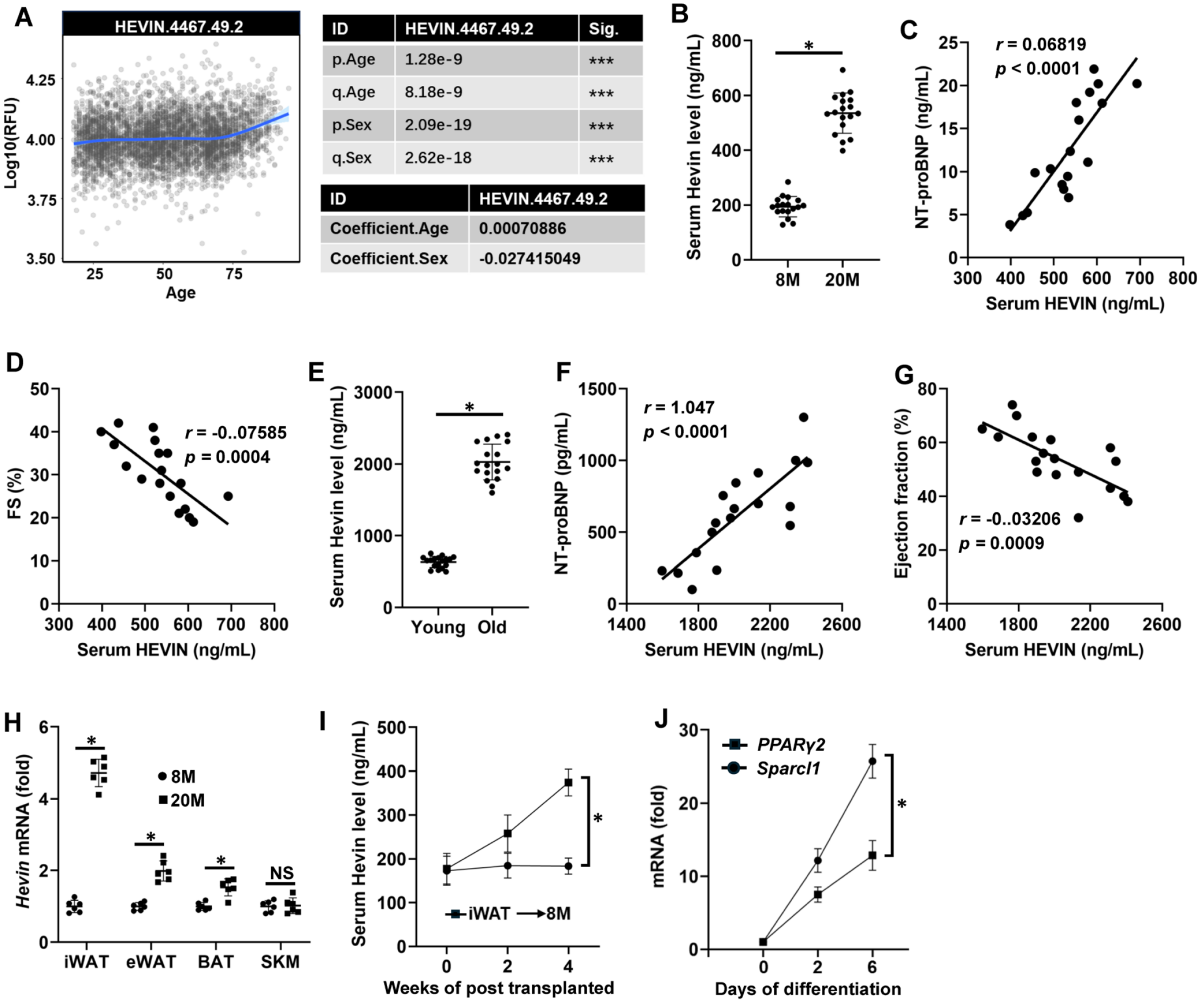
Hevin elevates in aging mouse circulation and correlates with cardiac function. (A) Human circulating Hevin levels were measured from young adults to nonagenarians with the SomaScan aptamer technology and analyzed using a public database. (B) Serum Hevin levels were measured in young and aging mice using a commercial kit (*n* = 18). (C, D) Pearson's correlation between serum Hevin levels and serum NT‐proBNP levels or FS in aging mice (*n* = 18). (E) Human circulating Hevin levels were measured from young participants (< 60 years old) and old participants (> 60 years old) using a commercial kit (*n* = 18). (F, G) Pearson's correlation between serum Hevin levels and serum NT‐proBNP levels or ejection fraction in old participants (*n* = 18). (H) mRNA level of Hevin in different organs in mice (*n* = 6). (I) Hevin expression in 3 T3‐L1 cells during differentiation (*n* = 6). All data are expressed as the mean ± S.D. and analyzed using one‐way ANOVA followed by Tukey post hoc test. *p < 0.05 versus the matched group.

To identify key targets involved in age‐related cardiac dysfunction, we initially searched a public database (https://twc‐stanford.shinyapps.io/aging_plasma_proteome/) featuring a human plasma proteome profile spanning the aging process, analyzed through the SomaScan aptamer technology and encompassing 22,925 plasma proteins, as outlined by Lehallier and colleagues (Lehallier et al. 2019). The notable increase of HEVIN in the circulation after the age of 70, as observed in the database, caught our attention (Figure 1A). Previous research has indicated that Hevin plays a crucial role in abnormal brain functional structure during the aging process (Seddighi et al. 2018). Additionally, Hevin has been established as a biomarker for right heart failure (di Salvo et al. 2015). Consequently, Hevin levels were found to be increased in the plasma of aging mice in contrast to their younger counterparts (Figure 1B). To further investigate whether the levels of Hevin in circulation are associated with age‐related cardiac dysfunction, we examined the relationship between Hevin levels in the circulation of aging mice and markers of heart failure. As shown in Figure 1C,D, serum Hevin levels in aging mice positively correlated with the levels of serum NT‐proBNP, a biomarker of cardiac dysfunction, but negatively correlated with fractional shortening (FS). Of note, compared with young people (< 60 years old), the elderly over 60 years old showed a notable increase in serum Hevin levels (Figure 1E). Consistently, the elevated serum Hevin in the elderly was associated with an increase in serum NT‐proBNP levels and a decline in ejection fraction (Figure 1F,G). We firstly examined whether the elevated Hevin in circulation originated from cardiac tissues. However, experimental results indicated no significant differences in Hevin expression in the hearts of aging mice compared to young mice (Figure S1A,B). Next, we investigated the expression changes of Hevin in inguinal white adipose tissue (iWAT), epididymal white adipose tissue (eWAT), brown adipose tissue (BAT), and skeletal muscle (SKM), where Hevin was found to be abundantly present (Liu et al. 2021). As described in Figure 1H, the expression of Hevin in the adipose tissue of aged mice significantly increased, particularly in the iWAT. To confirm that the increased Hevin in the circulation of aged mice originates from adipose tissue, we transplanted adipose tissues from different locations of aged mice into adult mice. The results showed a significant increase in circulating Hevin in adult mice 4 weeks after iWAT transplantation, while transplantation of eWAT or BAT did not increase Hevin levels in the circulation of mice (Figure 1I and Figure S1C,D). Moreover, it was found that the expression level of Hevin was highly induced along with PPARγ2 (an established adipogenic marker) during the differentiation of 3 T3‐L1 cells induced in vitro (Figure 1J). These findings suggest that aged mice adipose tissue secretes a significant amount of Hevin into circulation, and the circulating levels of Hevin are closely associated with age‐related decreases in cardiac FS and increases in NT‐proBNP.

### 3.2 Exposure to Hevin Promotes Aging‐Related Cardiac Inflammation and Dysfunction

**FIGURE 2:**
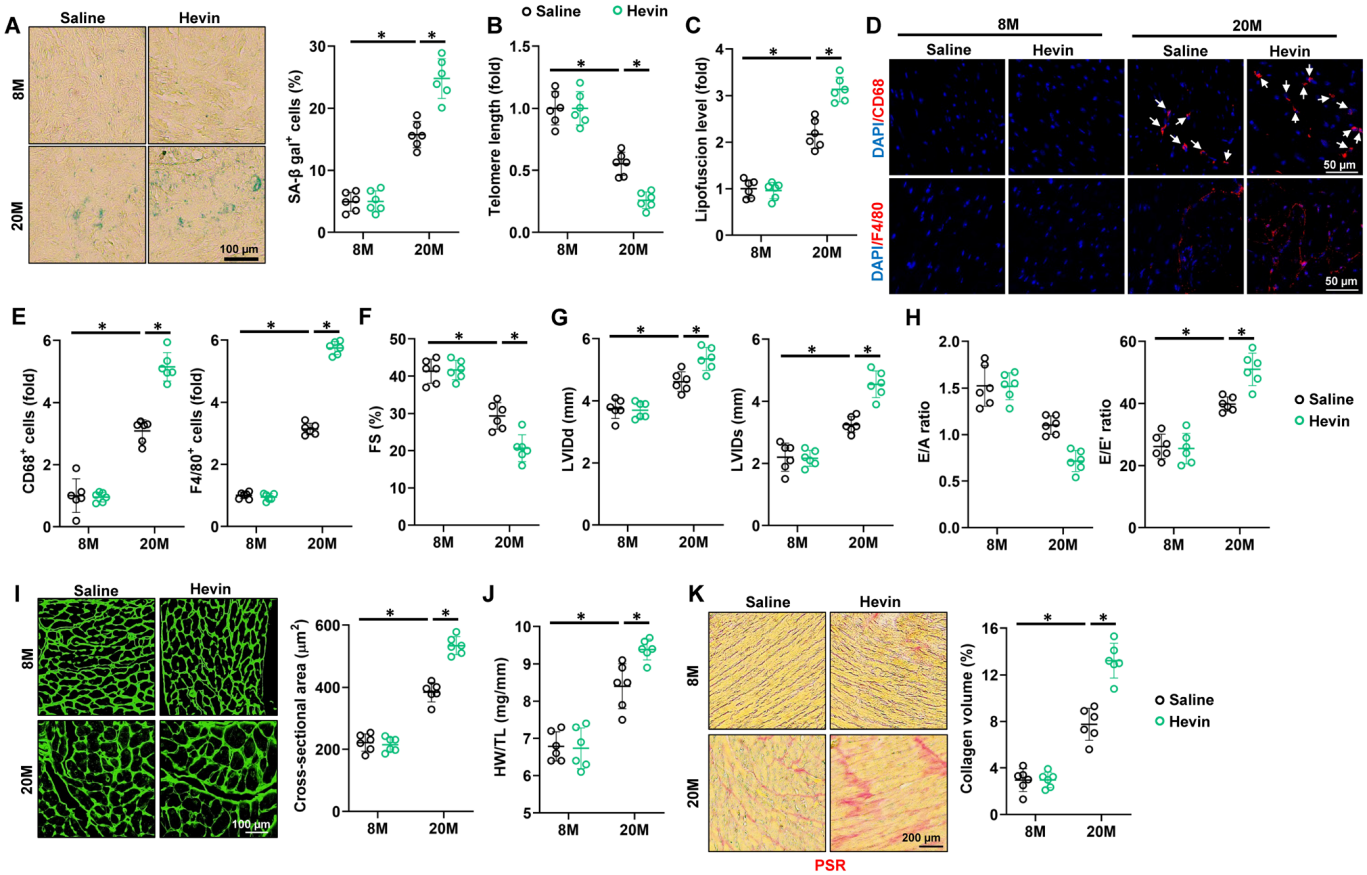
Exposure to Hevin promotes aging‐related cardiac inflammation and dysfunction. (A) Representative images and quantitative results of SA‐β gal staining in hearts from young and aging mice with or without Hevin administration (*n* = 6). (B) Relative telomere length in hearts (*n* = 6). (C) Relative lipofuscin accumulation in hearts (*n* = 6). (D‐E) The immunofluorescent staining and quantitative results of CD68 and F4/80 in murine hearts (*n* = 6). (F‐G) Echocardiographic and hemodynamic parameters of cardiac function in mice, including FS, LVIDd and LVIDs in mice (*n* = 6). (H) Tissue Doppler imaging was employed to measure E/A and E/E' to evaluate the diastolic function (*n* = 6). (I) Representative images and quantitative results of WGA staining in hearts (*n* = 6). (J) Heart weight‐to‐Tibia length (HW/TL) in mice (*n* = 6). (K) Representative images and quantitative results of PSR staining in hearts (*n* = 6). All data are expressed as the mean ± S.D., and analyzed using one‐way ANOVA followed by Tukey post hoc test. *p < 0.05 versus the matched group.

To study Hevin function during cardiac aging, aging and paired mice were intraperitoneally injected with saline or recombinant Hevin protein (0.2 mg/kg) every other day for 3 weeks. As shown in Figure S2A, Hevin administration significantly increased the serum Hevin level in mice. Hevin treatment did not alter mean arterial pressure (MAP) and heart rates in aged and matched mice (Figure S2B,C). Meanwhile, administration of Hevin did not lead to hepatic or muscular injuries, but worsening renal function during aging (Figure S2D,F). As shown in Figure 2A, Hevin injection dramatically promoted cellular senescence in aging heart. Meanwhile, the decreased telomere length and increased lipofuscin accumulation in aging hearts were intensified by Hevin administration (Figure 2B,C). Accordingly, Hevin injection also upregulated the expression of p16, p19, and p21 (Figure S3A,B). Recent findings have highlighted a direct link between low‐grade chronic inflammation and age‐related cardiac dysfunction (Liberale et al. 2020). As demonstrated in Figure S3C,D, IL‐6 and TNF‐α levels were elevated in aging hearts, whereas their concentrations further increased in hearts following Hevin administration. Immunohistochemistry staining indicated increased macrophage infiltration into murine hearts during the aging process, a phenomenon significantly exacerbated by Hevin injection (Figure 2D,E). Furthermore, Hevin administration further increased the levels of IL‐1β and IL‐18 in aging hearts (Figure S3E,F). As depicted in Figure 2F,G, Hevin administration significantly exacerbated the systolic function observed in aging hearts, demonstrated by deteriorations in fractional shortening (FS), left ventricular internal diameter in diastole (LVIDd), and left ventricular internal diameter in systole (LVIDs). Diastolic dysfunction is a key feature of cardiac aging, and our study revealed that aging mice administered Hevin displayed weakened diastolic function, as indicated by a decreased E/A ratio and increased E/E' ratio (Figure 2H). Cardiac hypertrophy and interstitial fibrosis are pivotal characteristics and factors contributing to age‐associated cardiac impairment (Hu, Zhang, Gao, et al. 2024). The cross‐sectional area of cardiomyocytes and the heart weight‐to‐tibia length (HW/TL) ratio increased in aging mice, with Hevin injection further augmenting both the cardiomyocytes cross‐sectional area and HW/TL ratio (Figure 2I,J). Meanwhile, the mRNA levels of hypertrophic markers in aging hearts were further disturbed by Hevin administration (Figure S3G). Hevin injection also exacerbated the collagen deposition in aging, accompanied by an elevation in the expression of fibrotic markers (Figure 2K and Figure S3H). Our findings indicate that chronic exposure to Hevin facilitates aging‐related cardiac inflammation and dysfunction.

Furthermore, we performed a single intraperitoneal injection of Hevin (0.2 mg/kg) to mimic the rapid increase of Hevin in the serum of individuals over 70 years old and study its effects on age‐related cardiac dysfunction (Figure 1A). As a result, acute Hevin treatment led to an increase in SA‐β gal^+^ cells in aging hearts (Figure S4A). Besides, the decreased telomere length and the increased lipofuscin accumulation caused by aging were exacerbated by acute Hevin injection (Figure S4B,C). Meanwhile, acute Hevin administration further aggravated aging‐related inflammation in the myocardium (Figure S4D,E). The age‐related decline in both systolic and diastolic function was significantly exacerbated in mice following acute Hevin injection, as demonstrated by elevated LVIDd and LVIDs, reduced FS, and altered E/A ratio (Figure S4F–H). Moreover, acute Hevin treatment markedly intensified age‐related cardiomyocyte hypertrophy and collagen deposition, indicated by the amplified cross‐sectional area of cardiomyocytes and collagen volume (Figure S4I,J). Collectively, these results suggest that acute Hevin injection can activate the aging‐related cardiac inflammation and dysfunction.

### 3.3 Hevin Knockout Alleviates Aging‐Related Cardiac Inflammation and Dysfunction

**FIGURE 3:**
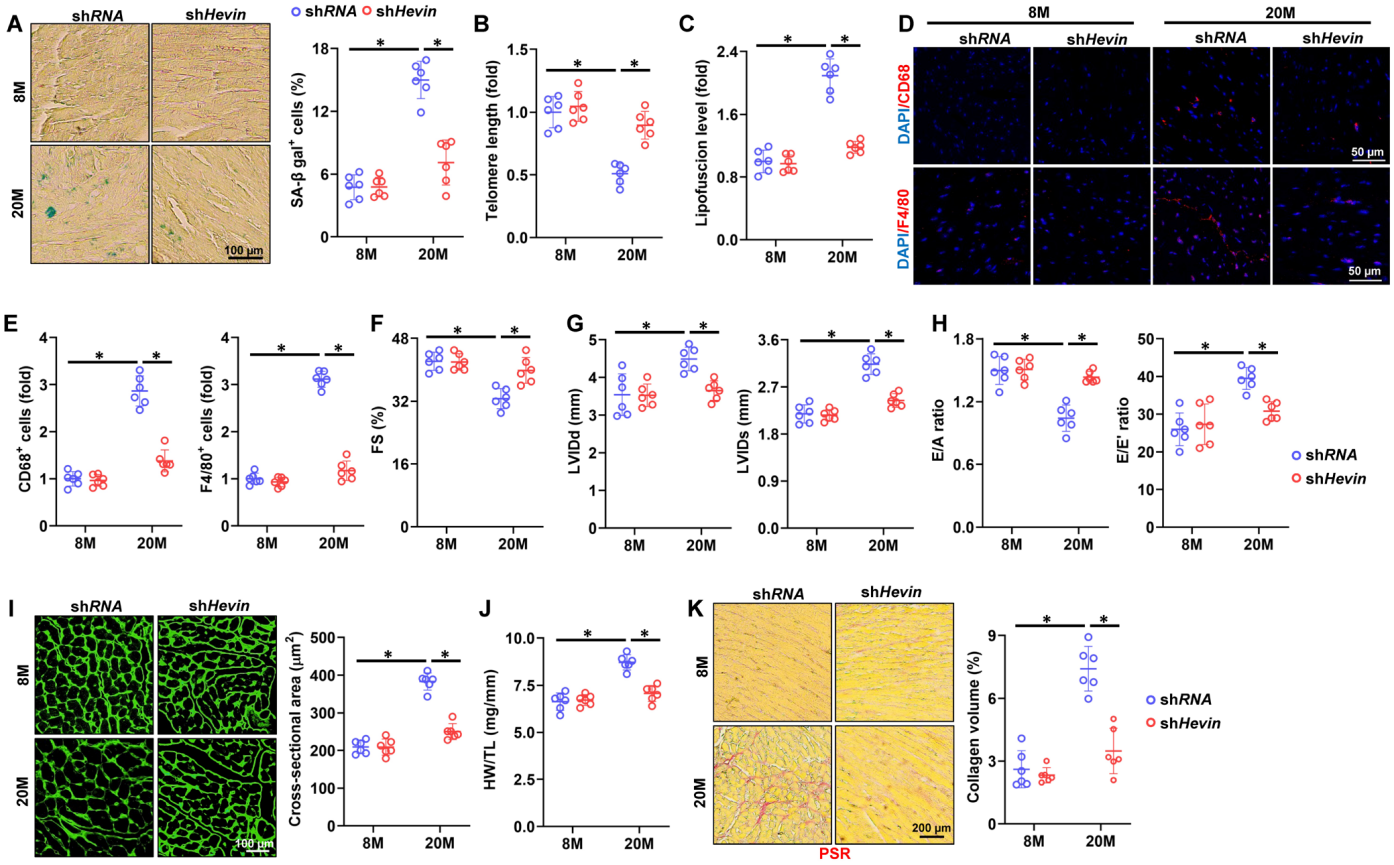
Hevin knockout alleviates aging‐related cardiac inflammation and dysfunction. (A) Representative images and quantitative results of SA‐β gal staining in hearts from young and aging mice (*n* = 6). (B) Relative telomere length in hearts (*n* = 6). (C) Relative lipofuscin accumulation in hearts (*n* = 6). (D‐E) The immunofluorescent staining and quantitative results of CD68 and F4/80 in murine hearts (*n* = 6). (F‐G) Echocardiographic and hemodynamic parameters of cardiac function in mice, including FS, LVIDd and LVIDs in mice (*n* = 6). (H) Tissue Doppler imaging was employed to measure E/A and E/E' to evaluate the diastolic function (*n* = 6). (I) Representative images and quantitative results of WGA staining in hearts (*n* = 6). (J) HW/TL ratio in mice (*n* = 6). (K) Representative images and quantitative results of PSR staining in hearts (*n* = 6). All data are expressed as the mean ± S.D., and analyzed using one‐way ANOVA followed by Tukey post hoc test. *p < 0.05 versus the matched group.

To delve deeper into the function of Hevin, we employed AAV vectors to knock down systemic expression of Hevin in mice. As illustrated in Figure S5A,B, the non‐specific systemic knockdown of Hevin in mice led to a notable decrease in Hevin levels in the serum, likely attributable primarily to the reduction of Hevin protein in iWAT. Specifically, Hevin knockdown did not affect MAP and heart rate in heart (Figure S5C,D). Hevin knockdown not only reduced the proportion of SA‐β ga^+^ senescent cells in aging hearts but also led to decreased lipofuscin accumulation and increased telomere length (Figure 3A–C). Moreover, the downregulation of Hevin resulted in decreased expression levels of p16, p19, and p21 (Figure S6A). Accordingly, the levels of IL‐6 and TNF‐α in the aging hearts significantly decreased following Hevin knockdown (Figure S6B,C). Macrophage infiltration also significantly improved following Hevin knockdown, accompanied by reductions in IL‐18 and IL‐1β levels (Figure 3D,E and Figure S6D,E). Consistent with expectations, knockdown of Hevin significantly relieved systolic and diastolic dysfunction in aging hearts (Figure 3F–H). Simultaneously, the knockdown of Hevin led to significant alleviation of age‐related cardiomyocyte hypertrophy and collagen deposition, as evidenced by the decreased cross‐sectional area of cardiomyocytes, HW/TL ratio, and collagen volume (Figure 3I–K). Hevin knockdown further reduced the mRNA levels of hypertrophic and fibrotic markers in aging hearts (Figure S6F,G). To further confirm the role of Hevin, we employed a separate shHevin# to validate specificity and eliminate any potential off‐target effects. As depicted in Figure S7A–C, cell senescence was ameliorated in shHevin#‐injected aging heart. Mice injected with shHevin# showed improvements in aging‐related systolic and diastolic dysfunction, demonstrated by reduced LVIDd and LVIDs, increased FS, and E/A ratios. Notably, as anticipated, aged mice receiving shHevin# injections exhibited reduced cardiomyocyte hypertrophy and interstitial fibrosis (Figure S7D–I). Our results suggest that Hevin knockout improves age‐related cardiac remodeling and dysfunction.

### 3.4 Hevin Exacerbates Age‐Related Cardiac Inflammation and Dysfunction by Inducing CCL5

**FIGURE 4:**
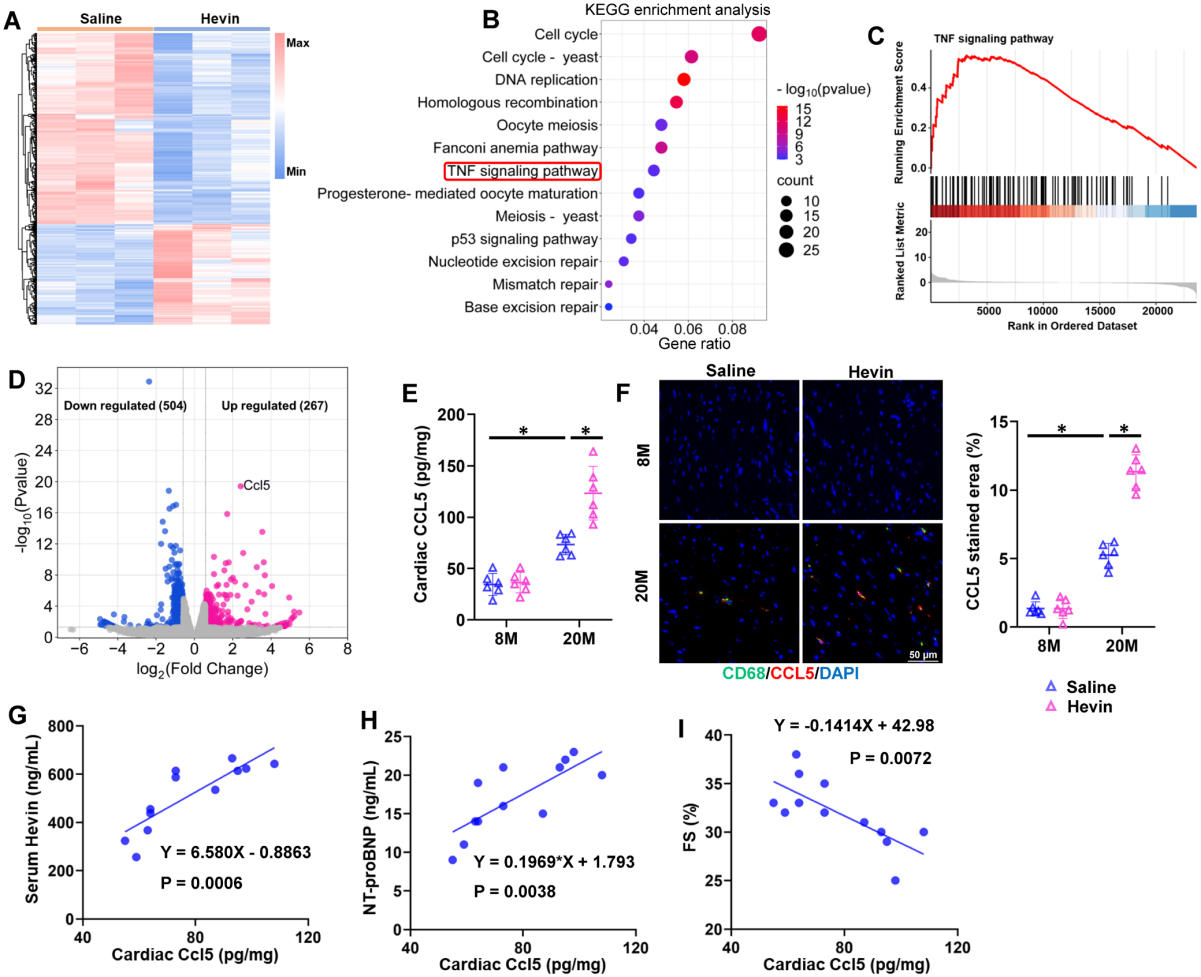
Hevin administration stimulates CCL5 expression. (A) Heart samples from aging mice with Hevin or saline administration based on RNA‐seq analysis. (B) KEGG pathway analysis of RNA‐seq. (C) GSEA of TNF signaling pathway. (D) Volcano map of significantly different genes in the heart tissues from mice in 20 M with Hevin or saline administration based on RNA‐seq analysis. (E) Cardiac CCL5 levels were measured in mice hearts using a commercial kit (*n* = 6). (F) Representative images and quantitative results of CCL5 staining in hearts (*n* = 6). (G) Pearson's correlation between serum Hevin levels and cardiac CCL5 levels in aging mice with Hevin administration (*n* = 12). (H) Pearson's correlation between serum NT‐proBNP levels and cardiac CCL5 levels in aging mice with Hevin administration (*n* = 12). (I) Pearson's correlation between FS and cardiac CCL5 levels in aging mice with Hevin administration (*n* = 12). All data are expressed as the mean ± S.D., and analyzed using one‐way ANOVA followed by Tukey post hoc test. *p < 0.05 versus the matched group.

**FIGURE 5:**
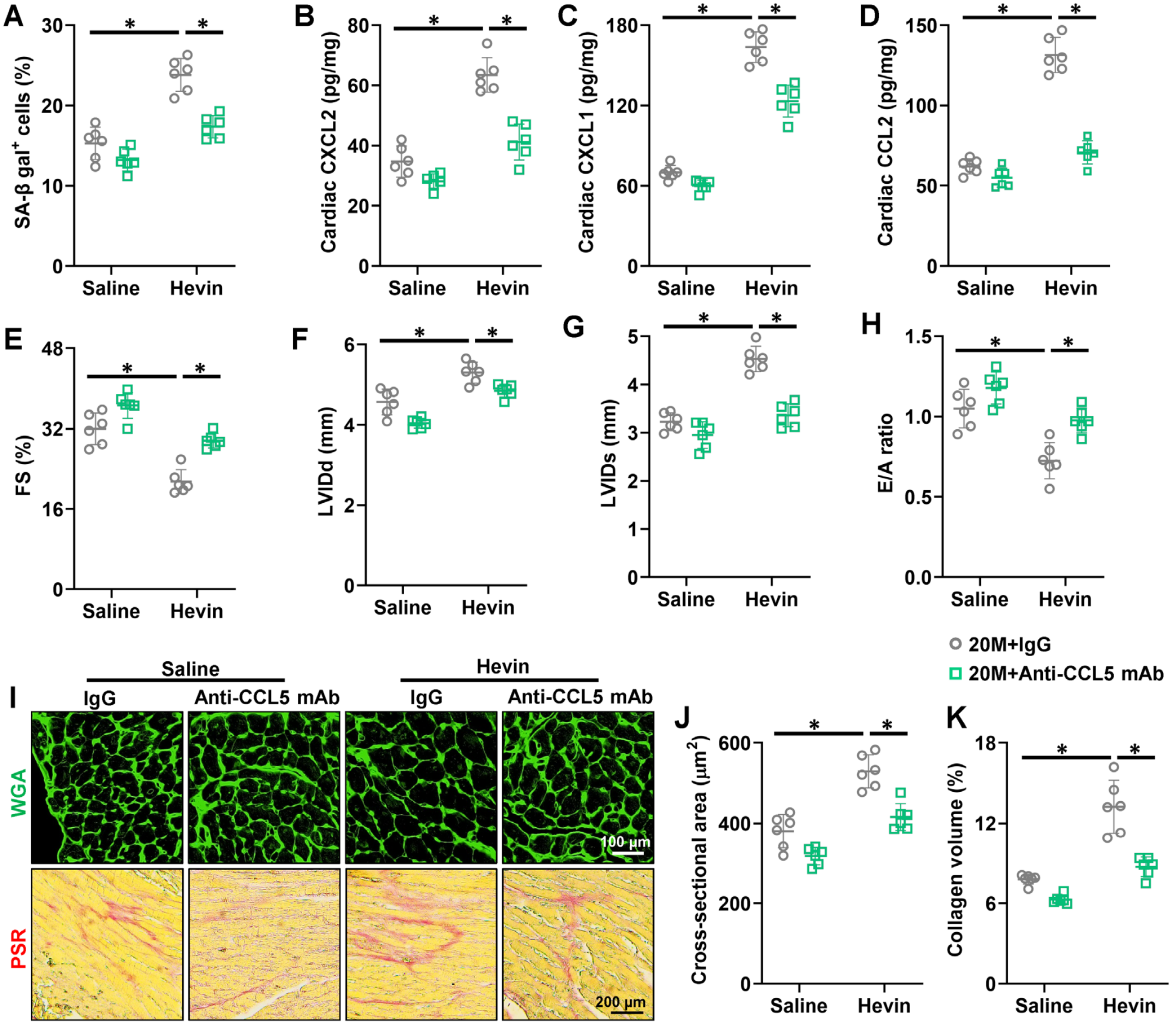
Hevin exacerbates age‐related cardiac inflammation and dysfunction by inducing CCL5. (A) Quantitative results of SA‐β gal staining in hearts from aging mice (*n* = 6). (B) Cardiac CXCL2 levels were measured in mice hearts using a commercial kit (*n* = 6). (C) Cardiac CXCL1 levels were measured in mice hearts using a commercial kit (*n* = 6). (D) Cardiac CCL2 levels were measured in mice hearts using a commercial kit (*n* = 6). (E‐G) Echocardiographic and hemodynamic parameters of cardiac function in mice, including FS, LVIDd, and LVIDs in mice (*n* = 6). (H) Tissue Doppler imaging was employed to measure E/A to evaluate the diastolic function (*n* = 6). (I‐K) Representative images and quantitative results of WGA and PSR staining in hearts (*n* = 6, differences between saline groups were not statistically evaluated). All data are expressed as the mean ± S.D., and analyzed using one‐way ANOVA followed by Tukey post hoc test. *p < 0.05 versus the matched group.

To uncover the molecular mechanisms underlying Hevin‐induced progression of age‐related cardiac dysfunction, we performed RNA‐Seq analysis to identify differentially expressed genes (DEGs) in the hearts of mice treated with chronic recombinant Hevin protein or saline, and the heatmaps of DEGs were provided in Figure 4A. Within the KEGG‐upregulated pathways induced by Hevin, the TNF signaling pathway has drawn our attention, as it plays a pivotal role in various cardiac dysfunctions (Figure 4B) (Feldman et al. 2000) Further validation through Gene set enrichment analysis (GSEA) confirmed a significant upregulation of the TNF signaling pathway induced by Hevin injection (Figure 4C). Besides, Hevin injection leads to a significant increase in various inflammation‐related pathways following Hevin injection, including the cytokine‐cytokine signaling pathway and the Toll‐like receptor signaling pathway (Figure S8A). Furthermore, GSEA further confirmed the upregulation of these pathways (Figure S8B,C). As shown in Figure 4D, 267 genes were upregulated and 504 genes were downregulated (fold change > 1.5, *p <* 0.05). Among them, we identified a pronounced overexpression of CCL5, a member of the C‐C chemokine family that is secreted by macrophages and directly activates M1 polarization while impeding M2 polarization. (Figure 4D and Figure S8D; Liu et al. 2020; Liu et al. 2024) In addition, our RNA‐Seq and ELISA analysis found that CXCL1, CXCL2, and CCL2 were also significantly upregulated in the hearts of Hevin‐treated mice (Figure S8D and Figure 5B–D). Matrix metalloproteinase 9 (MMP9), a protease involved in myocardial remodeling, was significantly upregulated in the hearts of aging mice following Hevin treatment (Figure S8F; Montecucco et al. 2012) The elevated levels of CCL5 in the hearts were validated through qRT‐PCR and ELISA analyses (Figure 4E and Figure S8E). Furthermore, immunostaining of CCL5 on mouse heart sections further confirmed an increase in CCL5 content in the aging heart following Hevin treatment (Figure 4F). Importantly, serum Hevin and cardiac CCL5 levels were positively correlated in aging mice (Figure 4G). In agreement, the elevated cardiac CCL5 levels in aging hearts were associated with an anticipated rise in plasma NT‐proBNP and a decline in FS (Figure 4H,I). To understand the reason that cell cycle is increased with Hevin treatment (Figure 4B). We performed co‐immunostaining of the proliferation marker Ki67 with the cardiomyocyte marker α‐actinin and the fibroblast marker Vimentin. The results showed that Hevin treatment markedly increased the number of Ki67‐positive cells in the heart, which is consistent with the upregulation of the cell cycle observed in the sequencing analysis. Moreover, Ki67 signals were found to colocalize with Vimentin, suggesting that the activation of cell cycle‐related pathways may be associated with enhanced fibroblast proliferation and increased fibrotic deposition in the aged heart following Hevin treatment (Figure S8G).

In order to investigate the involvement of CCL5 in mediating Hevin's impact on aging‐related cardiac dysfunction, we used 2 approaches to block CCL5 function, following which the effects of Hevin were assessed. First, we used Anti‐CCL5 mAb to inhibit its function in the hearts. As described in Figure 5A and Figure S9A,B, Anti‐CCL5 mAb significantly reversed the increased senescent cells post‐Hevin injection and restored the decreased telomere length and increased lipofuscin accumulation. Moreover, the upregulation of inflammatory factors such as CXCL1, CXCL2, and CCL2 induced by Hevin was also suppressed by Anti‐CCL5 mAb (Figure 5B–D). Consistent with the above findings, inflammatory markers induced by Hevin such as IL‐1β, IL‐6, IL‐18, and TNF‐α decreased following Anti‐CCL5 mAb treatment (Figure S9C–F). Accordingly, the Hevin‐induced deterioration of cardiac function associated with aging was attenuated following treatment with an Anti‐CCL5 mAb (Figure 5E–H). Histological examination demonstrated that Anti‐CCL5 mAb reversed Hevin‐associated hypertrophic growth and interstitial fibrosis in aged hearts (Figure 5I–K). Moreover, aged hearts with Anti‐CCL5 mAb exhibited decreased levels of *Anp*, *β‐Mhc*, *Col1α1*, *Col3α1*, and increased levels of *α‐Mhc* mRNA (Figure S9G,H). In addition, UK‐427857 (inhibitor of CCL5) was performed. As a result, the pathogenic roles of Hevin in aging‐related cardiac dysfunction were largely attenuated by UK‐427857 (Figure S10). Collectively, these results indicate that Hevin plays a role in the advancement of age‐related cardiac dysfunction, partly by triggering the induction of CCL5.

### 3.5 CCL5 Promotes Macrophage Polarization in the Aging Heart

**FIGURE 6:**
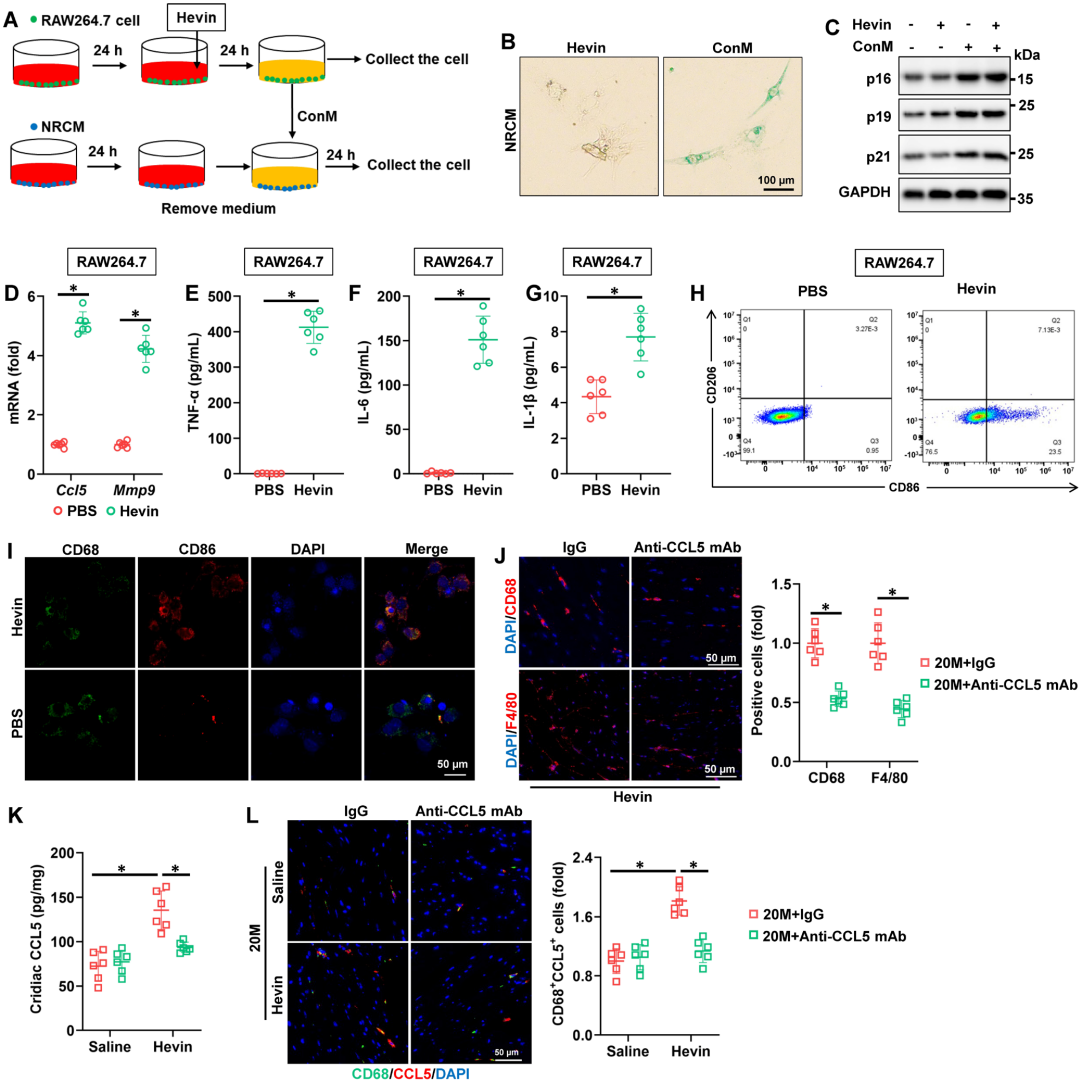
CCL5 promotes macrophage polarization in the aging heart. (A) Schematic protocol for Hevin treatment in vitro. (B) Representative pictures of SA‐β gal‐stained cell (*n* = 6). (C) Western blot images of p16, p19, and p21 in NRCMs (*n* = 6). (D) Relative *Ccl5* and *Mmp9* mRNA levels in RAW264.7 (*n* = 6). (E‐G) TNF‐α, IL‐6, and IL‐1β levels were measured in RAW264.7 using a commercial kit (*n* = 6). (H) Representative gating scheme for identification of M1‐like (CD86 + CD206−) and M2‐like (CD86 − CD206+) macrophages in RAW264.7 (*n* = 6). (I) Representative image of CD86 and CD68 staining in RAW264.7 (*n* = 6). (J) Representative image of CD68 and F4/80 staining in hearts (*n* = 6). (K) Cardiac CCL5 levels were measured in mice hearts using a commercial kit (*n* = 6). (L) The immunofluorescent staining and quantitative results of CD68 + CCL5+ cells in murine hearts (*n* = 6). All data are expressed as the mean ± S.D., and analyzed using one‐way ANOVA followed by Tukey post hoc test. *p < 0.05 versus the matched group.

To further investigate the role of CCL5 in Hevin‐mediated age‐related cardiac dysfunction, we first explored the source of CCL5 in the heart. As shown in Figure S11A, following Hevin treatment, there was a significant increase in the expression of CCL5 in cardiac macrophages of aged mice. Correspondingly, the expression of MMP9 in macrophages was also significantly upregulated after Hevin injection (Figure S11B). In order to investigate whether CCL5 secreted by cardiac macrophages plays a crucial role, we treated RAW264.7 cells with Hevin in vitro and used the conditioned media (ConM) to treat neonatal rat cardiomyocytes (NRCMs) (Figure 6A). As shown in Figure 6B, ConM significantly induced senescence in NRCM cells, while treatment with Hevin did not lead to senescence in NRCM cells. Meanwhile, treatment with ConM increased the expression of p16, p19, and p21 in NRCM (Figure 6C). More importantly, following Hevin treatment, there was a significant increase in the expression of CCL5 and MMP9 in RAW264.7 cells (Figure 6D). Furthermore, the expression of inflammatory factors, including TNF‐α, IL‐6, and IL‐1β, were elevated in RAW264.7 cells following Hevin treatment (Figure 6E–G). Besides, co‐culture of RAW264.7 cells and NRCMs with or without Hevin identified that co‐culture of RAW264.7 cells and NRCMs with Hevin induced NRCMs senescence (Figure S12A–C). These results suggest that the inflammatory factors secreted by Hevin‐stimulated macrophages promote senescence in NRCMs. Macrophage activation is increasingly recognized as a dynamic and plastic spectrum rather than a binary polarization state. Nevertheless, for descriptive and operational purposes, macrophage functional states are often characterized based on the predominance of pro‐inflammatory or anti‐inflammatory features. Macrophages exhibiting pro‐inflammatory characteristics are commonly referred to as M1‐like, whereas those displaying anti‐inflammatory and tissue‐remodeling properties are described as M2‐like phenotypes. Pro‐inflammatory (M1‐like) macrophages are associated with the initiation and amplification of inflammatory responses through the recruitment of immune cells and the production of inflammatory cytokines, whereas anti‐inflammatory (M2‐like) macrophages contribute to inflammation resolution and immune regulation (Kang et al. 2024; Wilson et al. 2018). Based on this framework, we next investigated whether Hevin modulates macrophage functional polarization. As shown in Figure 6H, flow cytometry analysis demonstrated that Hevin treatment increased the proportion of macrophages exhibiting M1‐like, pro‐inflammatory features. Consistently, immunofluorescence staining revealed an enrichment of M1‐associated characteristics following Hevin treatment, with a concomitant reduction in M2‐associated features (Figure 6I and Figure S11C). Furthermore, Hevin treatment upregulated the expression of pro‐inflammatory macrophage markers, including CD86, CD80, and CD32, while downregulating anti‐inflammatory macrophage‐associated markers such as CD206, CD163, and CD204 (Figure S11D–E). Inflammatory cytokines recruit inflammatory cells to inflamed sites; therefore, we investigated whether the increased inflammatory cells in the aging heart following Hevin treatment were recruited by CCL5. As depicted in Figure 6J, after Anti‐CCL5 mAb blocking of Hevin, the infiltration of inflammatory cells in the hearts of aged mice significantly decreased. Accordingly, the expression of CCL5 and MMP9 in macrophages in the aging heart also significantly decreased after Hevin blockade (Figure 6K,L and Figure S11F–G). To further enhance the rigor of our current study, we isolated monocytes from human peripheral blood and treated them with Hevin. The results showed that Hevin treatment increased the mRNA expression levels of CCL5 and MMP9 in monocytes (Figure S12D). Moreover, Hevin treatment induced the differentiation of monocytes into mature macrophages, as evidenced by elevated levels of TNF‐α and IL‐6 in the culture supernatant (Figure S12E). These findings are consistent with the results observed in RAW264.7 cells. Collectively, we demonstrated that Hevin stimulation promotes the secretion of CCL5 by cardiac macrophages, recruiting more inflammatory cells to the heart and facilitating the polarization of macrophages into the M1 phenotype, which secrete inflammatory factors to promote the aging of myocardial cells.

### 3.6 Hevin Regulates CCL5 Expression Through Binding to TLR4 and Activation of the p65

**FIGURE 7:**
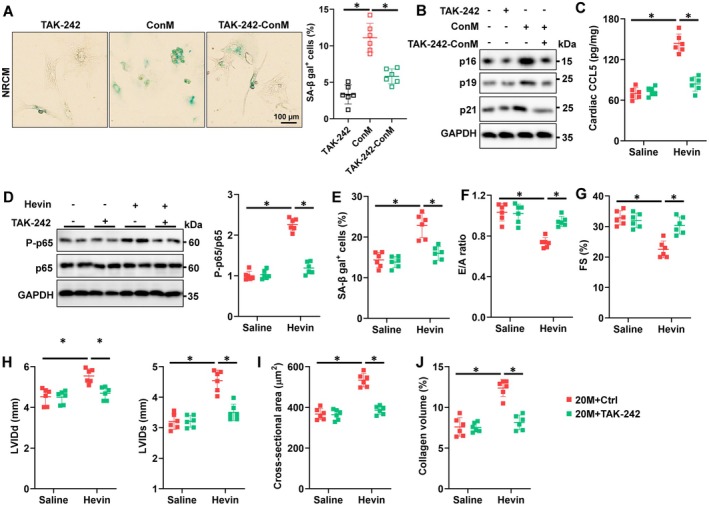
Hevin regulates CCL5 expression through binding to TLR4 and activation of the p65. (A) Representative pictures and quantitative results of SA‐β gal‐stained cells (*n* = 6). (B) Western blot images of p16, p19, and p21 in NRCMs (*n* = 6). (C) Cardiac CCL5 levels were measured in mice hearts using a commercial kit (*n* = 6). (D) Western blot images of p65 and P‐p65 in hearts (*n* = 6). (E) Quantitative results of SA‐β gal staining in hearts (*n* = 6). (F) Tissue doppler imaging was employed to measure E/A to evaluate the diastolic function (*n* = 6). (G‐H) Echocardiographic and hemodynamic parameters of cardiac function in mice, including FS, LVIDd, and LVIDs in mice (*n* = 6). (I, J) Quantitative results of WGA and PSR staining in hearts (*n* = 6). All data are expressed as the mean ± S.D., and analyzed using one‐way ANOVA followed by Tukey post hoc test. *p < 0.05 versus the matched group.

Next, we investigated the molecular basis by which Hevin upregulates CCL5 expression in macrophages. Previous studies have found that Hevin can activate NF‐κB/p65 through the TLR4 receptor and regulate the transcription of cytokines. Therefore, we used TAK‐242, a selective inhibitor of TLR4 signaling, to investigate whether Hevin exerts its effects through TLR4. As shown in Figure S13A, TAK‐242 blocked the stimulatory effect of Hevin on the expression of CCL5 and MMP9 in macrophages. The increase of TNF‐α, IL‐6, and IL‐1β in RAW264.7 cells following Hevin stimulation was also disturbed by TAK‐242 (Figure S13B–D). As expected, the TAK‐242‐ConM no longer promoted the senescence of NRCMs, accompanied by a reversal in the expression of p16, p19, and p21 (Figure 7A,B). Furthermore, in RAW264.7 cells, Hevin stimulation enhanced p65 activation, while TAK‐242 could block Hevin's activating effect on p65 (Figure S13E).

To further validate the crucial role of TLR4 in mediating the effects of Hevin in the aging heart in vivo, we initially examined the impact of intravenous injection of TAK‐242 on p65. As depicted in Figure 7C and Figure S13F,G, TAK‐242 blocked the upregulation of CCL5 and MMP9 induced by Hevin stimulation. Treatment with Hevin promoted the activation of P65 in cardiac tissues, while TAK‐242 inhibited this effect (Figure 7D). In addition, the accelerating effects of Hevin on aging‐related cellular senescence were offset by TAK‐242 (Figure 7E). Hevin failed to deteriorate aging‐induced inflammatory response in hearts with TAK‐242, as determined by the decreased levels of IL‐1β, IL‐18, IL‐6 and TNF‐α (Figure S13H–K). Furthermore, TAK‐242 administration in mice with Hevin‐injected aging hearts led to an amelioration of both systolic and diastolic functions (Figure 7F–H). Meanwhile, TAK‐242 treatment in mice counteracted the facilitating effects of Hevin on age‐related cardiac remodeling (Figure 7I,J). Lastly, to enhance the therapeutic effects of Hevin on age‐related cardiac dysfunction, we continuously fed aged mice after excision of their iWAT to observe if it could improve cardiac dysfunction. As shown in Figure S14A,B, a significant decrease in Hevin levels in the serum was observed after excision of iWAT, accompanied by a reduction of CCL5 levels in the hearts of aged mice. Accordingly, the age‐related elevation of SA‐β gal+ cells and inflammatory factors in the myocardium was likewise diminished through the removal of iWAT (Figure S14C–E). Similarly, the excision of iWAT improved the contractile and diastolic function of the hearts in aged mice (Figure S14F,G). Collectively, we conclude that Hevin promotes the transcription of CCL5 by activating P65 via TLR4, thereby exacerbating age‐related cardiac functional impairments.

## 4 Discussion

The process of aging stands as an autonomous determinant of cardiovascular diseases, with age‐related cardiac dysfunction playing a significant role in amplifying disability and mortality rates among the elderly (Triposkiadis et al. 2019). In our current investigation, we showcase an elevation of Hevin levels in the serum of aging mice, with a corresponding correlation between increased serum Hevin and diminished cardiac function. The administration of Hevin enhances these effects, whereas knockout of Hevin mitigates aging‐related cardiac remodeling and dysfunction. RNA‐seq analysis reveals that Hevin triggers the expression of CCL5 in aging hearts, and the inhibition of CCL5 nullifies the deleterious effects of Hevin‐induced cardiac aging in vivo. Mechanistically, Hevin released by iWAT into circulation stimulates cardiac macrophages through TLR4, inducing their polarization and CCL5 secretion, leading to worsened cardiac dysfunction and subsequent recruitment of additional inflammatory cells for pro‐inflammatory factor release. In aggregate, our discoveries underscore the pivotal role of Hevin in modulating inflammation throughout cardiac aging, representing the inaugural identification of Hevin as a prospective therapeutic target in the realm of cardiac aging. An important observation of this study is that Hevin overexpression alone does not induce overt cardiac inflammation or functional impairment in 8‐month‐old mice. This suggests that Hevin is not a primary initiator of cardiac pathology. Instead, its effects become evident in the context of an aged microenvironment, where baseline inflammatory signaling, immune cell priming, and extracellular matrix remodeling are already present. Under these conditions, Hevin likely acts as an additive or amplifying factor that exacerbates age‐associated inflammatory and functional decline.

Aging is characterized by a systematic decline in organ functions (Qiu et al. 2025). Adipose tissue can be broadly categorized as WAT or BAT, and unlike BAT, the presence and activity of WAT increase with age (Cypess et al. 2009; Saito et al. 2009; van Marken Lichtenbelt et al. 2009). WAT serves as the primary reservoir for lipid storage, but is also an active endocrine organ (Rogers et al. 2012). Increasing adiposity and ectopic fat accumulation contribute along to the development of inflammation in older humans (Barzilai et al. 2012; Jura and Kozak 2016; Kuk et al. 2009). Our study reveals elevated Hevin expression in aged mouse iWAT, released into circulation, while Liu et al. similarly report heightened Hevin expression in WAT post‐Nonalcoholic fatty liver disease (NAFLD), playing a crucial role in NAFLD pathogenesis. However, due to constraints, we did not validate the results using transgenic mice, which would enhance the accuracy of the findings. Additionally, we only validated in vitro that Hevin promotes macrophage polarization, though previous studies have also demonstrated Hevin's ability to induce macrophage polarization towards the M1 phenotype (Zhao et al. 2023, 2024). Our study reveals that Hevin promotes CCL5 expression and that blocking the action of CCL5 can reduce inflammatory cell infiltration in the heart, consistent with previous findings that CCL5 plays an active role in recruiting a variety of leukocytes into inflammatory sites including T cells, macrophages, eosinophils, and basophils (Lv et al. 2013). Furthermore, a wealth of prior research has demonstrated the significant role of CCL5 in various types of cardiac‐related diseases (Mikolajczyk et al. 2021; Wu et al. 2024; Wu et al. 2023). Of particular significance is our observation that the administration of Hevin exacerbates renal dysfunction in aged mice (Figure S2E). During physiological aging, kidneys experience detrimental structural and functional changes. Several renal pathologies such as acute kidney injury, glomerulonephritis, diabetic nephropathy, polycystic kidney disease, and chronic kidney disease (CKD) have been associated with cellular senescence and telomere dysfunction (Rossiello et al. 2022; Sturmlechner et al. 2017). This suggests that Hevin may play a crucial role in age‐induced renal dysfunction, necessitating further research to substantiate the role of Hevin in the kidney. Moreover, Figure 1A indicates a correlation between Hevin expression and gender, yet we did not assess the role of Hevin in female mice. The gender‐related disparities in Hevin expression may be attributed to variations in the content and activity of adipose tissue in different sexes (Bond et al. 2021; Vick et al. 2023). Our study revealed that the excision of iWAT in aged mice can delay the development of age‐related cardiac dysfunction, suggesting that pharmacological interventions targeting iWAT control could potentially ameliorate age‐related cardiac dysfunction. As individuals age, there is a natural decline in metabolic rate and muscle mass, which can contribute to weight gain and an increased risk of obesity (Kivimaki et al. 2024). The accumulation of visceral fat, especially around the abdomen, is particularly concerning as it is linked to inflammation, insulin resistance, and metabolic disturbances, all of which can impact overall health and exacerbate the aging process (Diaz‐Ruiz et al. 2023; Spinelli et al. 2023). There are now numerous medications available for weight loss, such as semaglutide, which has been shown to alleviate cardiac dysfunction (Davies et al. 2021; Deanfield et al. 2024; Kosiborod et al. 2024; Verma et al. 2024). However, further exploration is needed to determine whether semaglutide can exert beneficial effects in age‐related diseases by reducing the secretion of adipose tissue‐derived proteins such as Hevin.

In essence, our discoveries demonstrate that Hevin fosters age‐related cardiac dysfunction by activating cardiac macrophages via TLR4, prompting their polarization and CCL5 release. This cascade exacerbates cardiac dysfunction, triggering the influx of further inflammatory cells for the dissemination of pro‐inflammatory factors. Notably, our study identifies Hevin as a promising predictive and therapeutic target in the realm of cardiac aging.

## Author Contributions

Min Hu, Yu‐Jie Chen, and Shi‐Yu Huang designed the study and wrote the manuscript. Shi‐Yu Huang, Yu‐Jie Chen, and Min Hu performed animal and cell culture experiments. Yu‐Xin Hu and Jia‐Chen Liu participated in the interpretation of the results. Yu‐Xin Hu, Shi‐Yu Huang, and Min Hu finalized the manuscript. All authors critically read and commented on the final manuscript.

## Funding

This work was supported by grants from Undergraduate Training Programs for Innovation and Entrepreneurship of Wuhan University (202510486144, S202410486319, 202310486102).

## Conflicts of Interest

The authors declare no conflicts of interest.

## Supporting information


**Data S1:** acel70369‐sup‐0001‐DataS1.docx.

## Data Availability

The data that supports the findings of this study are available in the Supporting Information of this article.
