# Supplementary material for: Hevin Promotes Aging‐Related Cardiac Dysfunction via Facilitating Cardiac Inflammation in Male Mice
Source: Aging Cell. 2026 Jan 11;25(2):e70369. doi: 10.1111/acel.70369 (PMC12791026; doi:10.1111/acel.70369)
Supplement: Supplementary file 1 — Data S1: acel70369‐sup‐0001‐DataS1.docx. [file ACEL-25-e70369-s001.docx]

**Hevin promotes aging-related cardiac dysfunction via facilitating cardiac inflammation in male mice**

Shi-Yu Huang^1,2,3#^, Yu-Jie Chen^2#^, Yu-Xin Hu^4^, Jia-Chen Liu^2^, Min Hu^1,4^*

**Supplementary Table S1** The antibody used in the article

| REAGENT | SOURCE | IDENTIFIER |
| --- | --- | --- |
| p-P65 | Cell Signaling Technology | #3033 |
| t-P65 | Cell Signaling Technology | #8242 |
| GAPDH Rabbit mAb | Cell Signaling Technology | #2118 |
| AKT | Cell Signaling Technology | #4691 |
| p-AKT | Cell Signaling Technology | #4060 |
| p16 | Santa Cruz Biotechnology | sc-1661 |
| p19  p21  HEVIN | Santa Cruz Biotechnology  Santa Cruz Biotechnology  Abcam | sc-32748  sc-6246  ab313638 |

**Supplementary Table S2** The primers used in quantitative real-time PCR

| **Species** | **Gene** | **Forward primer** | **Reverse primer** |
| --- | --- | --- | --- |
| Mice  Mice  Mice  Mice  Mice  Mice  Mice  Mice | *Hevin*  *Anp*  *α-Mhc*  *β-Mhc*  *Col1α1*  *Col3α1*  *Il-6*  *Tnf-α* | CAGAGCAGCAGGACCAAGAA  ACCTGCTAGACCACCTGGAG  GGATGCCCTGCTGGTTA  CCGAGTCCCAGGTCAACAA  AGGCTTCAGTGGT T TGGATG  CCCAACCCAGAGATCCCATT  AGTTGCCTTCTTGGGACTGA  GCAAAGGGAGAGTGGTCA | TATCACCAGCTCCTCCGTGA  CCTTGGCTGTTATCTTCGGTACCGG  CGCCCAAACTCCTCCTT  CTTCACGGGCACCCTTGGA  CACCAACAGCACCATCGTTA  GAAGCACAGGAGCAGGTGTAGA  TCCACGATTTCCCAGAGAAC  CTGGCTCTGTGAGGAAGG |
| Mice | *Gapdh* | ACTCCACTCACGGCAAATTC | TCTCCATGGTGGTGACGACA |


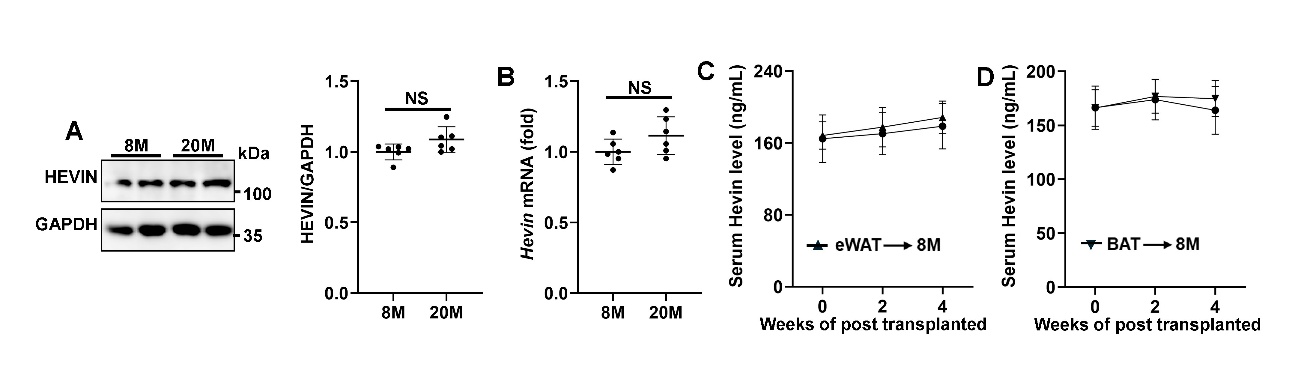


**Figure S1. Hevin elevates in aging mouse circulation and correlates with cardiac function.**

**(A)** Western blot images of HEVIN, and the statistical results (n = 6). **(B)** Relative *Hevin* mRNA levels in hearts (n = 6). **(C-E)** Serum Hevin levels in mice (n = 6). All data are expressed as the mean ± S.D., and analyzed using one-way ANOVA followed by Tukey post hoc test. **P*< 0.05versus the matched group.


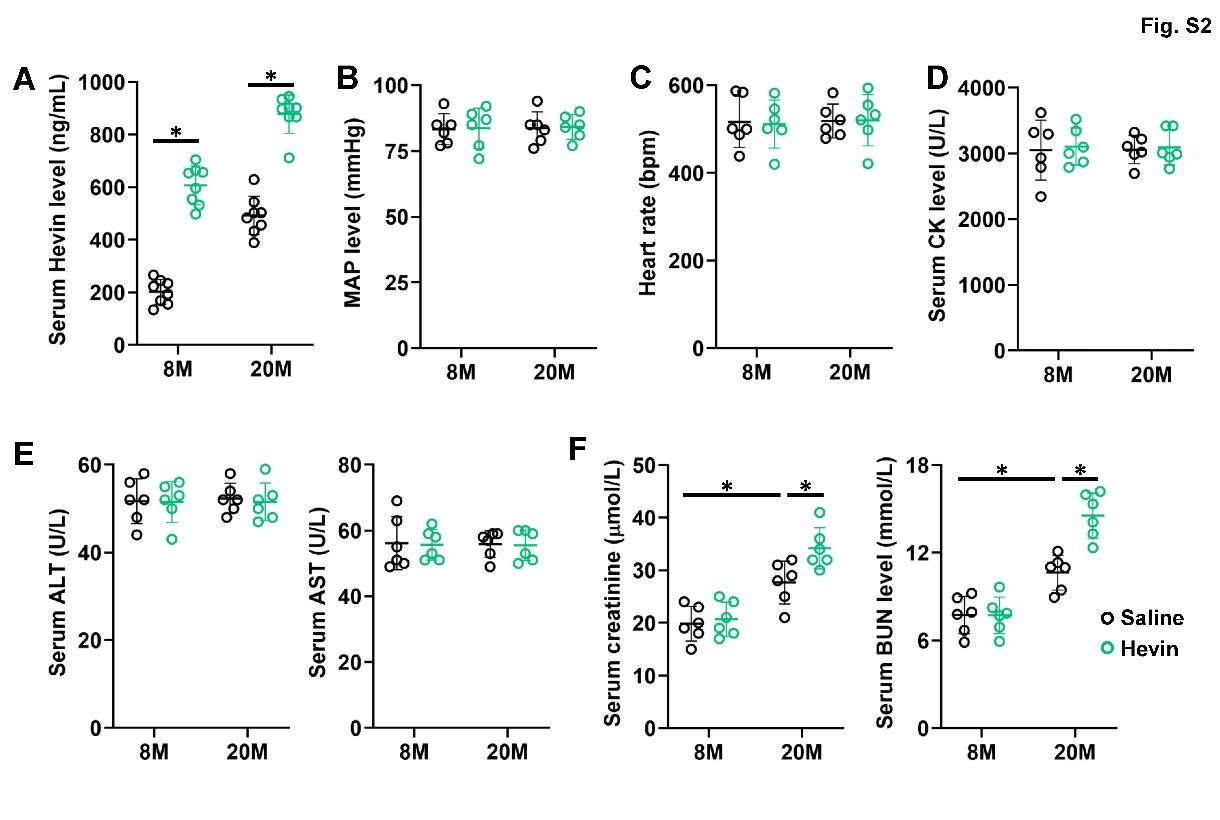


**Figure S2. Exposure to Hevin promotes aging-related cardiac inflammation and dysfunction.**

**(A)** Serum Hevin level after recombinant Hevin administration (n = 6). **(B)** MAP was determined in mice among groups (n = 6). **(C)** Heart rate among groups (n = 6). **(D)** Serum CK levels in mice (n = 6). **(E)** Serum liver enzymes levels (n = 6). **(F)** Serum creatinine and BUN levels (n = 6). All data are expressed as the mean ± S.D., and analyzed using one-way ANOVA followed by Tukey post hoc test. **P*< 0.05versus the matched group.


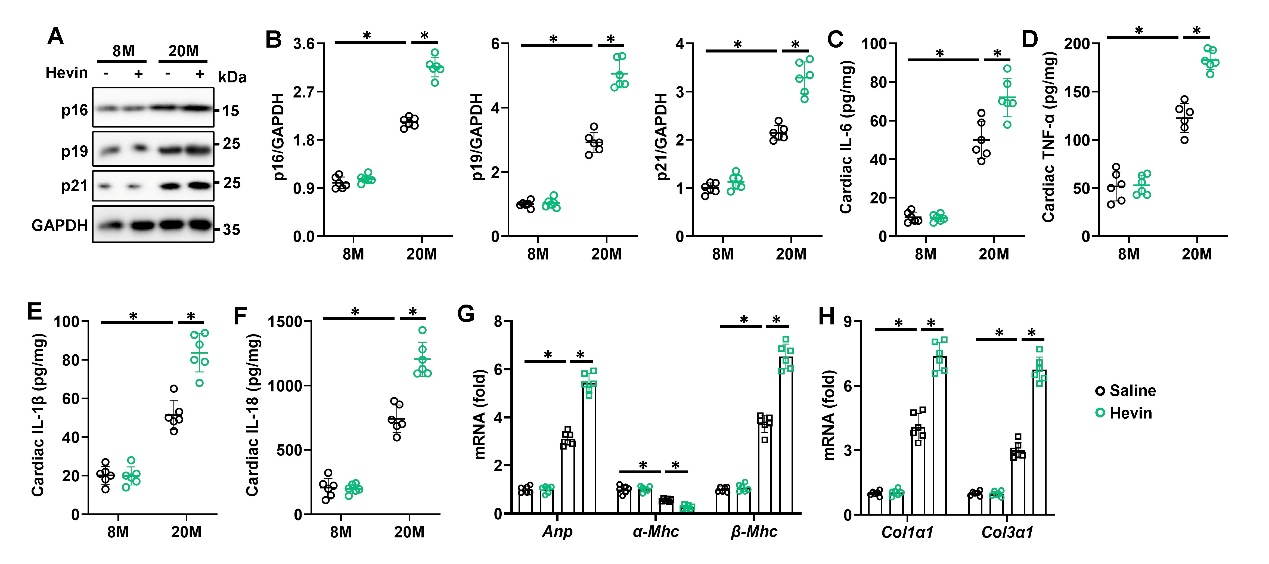


**Figure S3. Chronic exposure to Hevin promotes aging-related cardiac inflammation and dysfunction.**

**(A-B)** Western blot images of p16, p19, and p21, and the statistical results (n = 6). **(C-F)** The myocardial IL-6, TNF-α, IL-1β and IL-18 levels were determined by ELISA kits (n = 6). **(G)** Relative *Anp*, *α-Mhc* and *β-Mhc* mRNA levels in hearts (n = 6). **(H)** Relative *Col1α1* and *Col3α1* mRNA levels in hearts (n = 6). All data are expressed as the mean ± S.D., and analyzed using one-way ANOVA followed by Tukey post hoc test. **P*< 0.05versus the matched group.


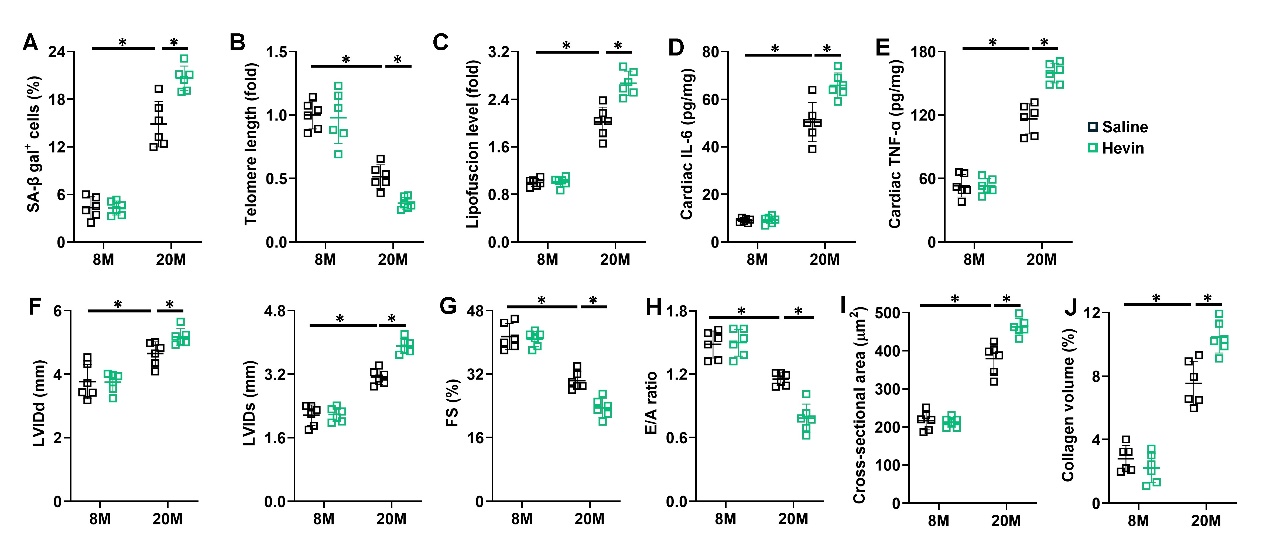


**Figure S4. Acute injection of Hevin promotes aging-related cardiac inflammation and dysfunction.**

**(A)** Quantitative results of SA-β gal staining in hearts from young and aging mice (n = 6). **(B)** Relative telomere length in hearts (n = 6). **(C)** Relative lipofuscin accumulation in hearts (n = 6). **(D-E)** The myocardial IL-6 and TNF-α levels were determined by ELISA kits (n = 6). **(F-G)** Echocardiographic and hemodynamic parameters of cardiac function in mice, including FS, LVIDd and LVIDs in mice (n = 6). **(H)** Tissue Doppler imaging was employed to measure E/A to evaluate the diastolic function (n = 6). **(I-J)** Quantitative results of WGA and PSR staining in hearts (n = 6). All data are expressed as the mean ± S.D., and analyzed using one-way ANOVA followed by Tukey post hoc test. **P*< 0.05versus the matched group.


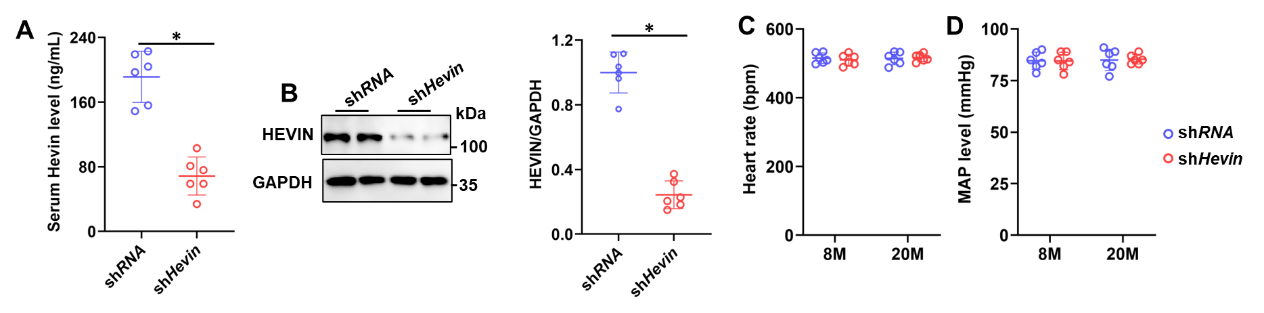


**Figure S5. Sh*Hevin* effective knock-down Hevin in mice**

**(A)** Serum Hevin levels in mice (n = 6). **(B)** Western blot images of Hevin and the statistical results (n = 6). **(C)** Heart rate among groups (n = 6). **(D)** MAP was determined in mice among groups (n = 6). All data are expressed as the mean ± S.D., and analyzed using one-way ANOVA followed by Tukey post hoc test. **P*< 0.05versus the matched group.


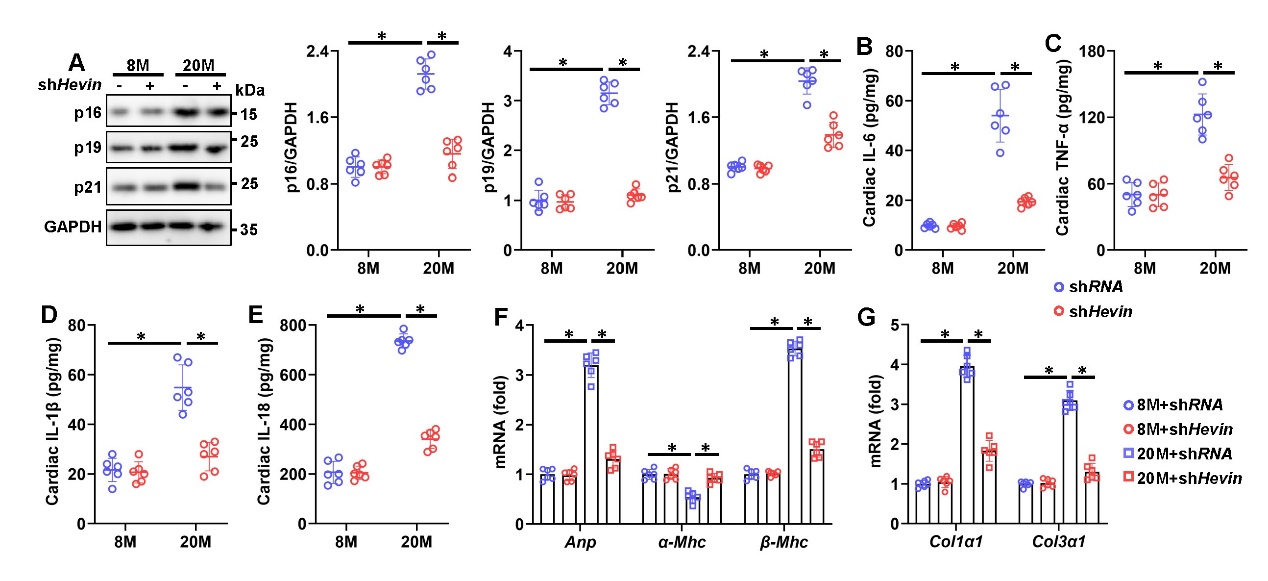


**Figure S6. Hevin knockout alleviates aging-related cardiac inflammation and dysfunction.**

**(A)** Western blot images of p16, p19, and p21, and the statistical results (n = 6). **(B-E)** The myocardial IL-6, TNF-α, IL-1β and IL-18 levels were determined by ELISA kits (n = 6). **(F)** Relative *Anp*, *α-Mhc* and *β-Mhc* mRNA levels in hearts (n = 6). **(G)** Relative *Col1α1* and *Col3α1* mRNA levels in hearts (n = 6). All data are expressed as the mean ± S.D., and analyzed using one-way ANOVA followed by Tukey post hoc test. **P*< 0.05versus the matched group.


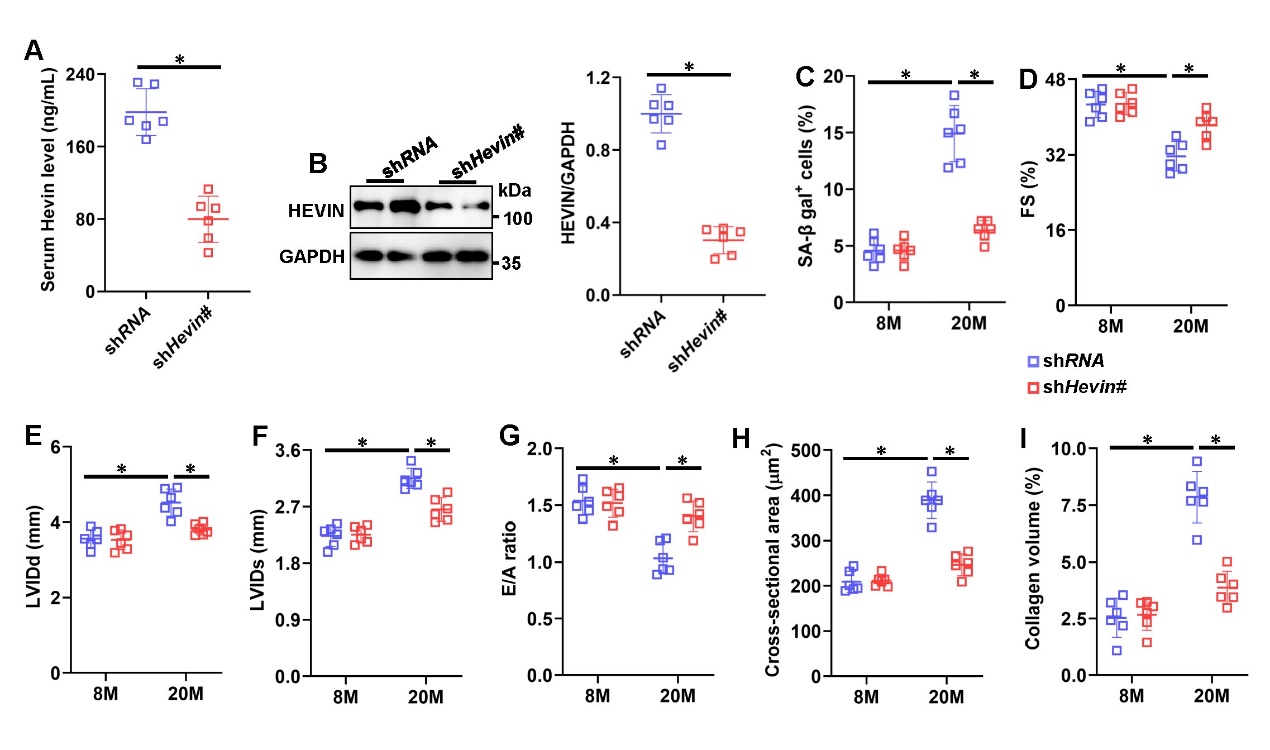


**Figure S7. Hevin knockout with sh*Hevin#* alleviates aging-related cardiac inflammation and dysfunction.**

**(A)** Serum Hevin levels in mice (n = 6). **(B)** Western blot images of Hevin and the statistical results (n = 6). **(C)** Quantitative results of SA-β gal staining in hearts from young and aging mice (n = 6). **(D-F)** Echocardiographic and hemodynamic parameters of cardiac function in mice, including FS, LVIDd and LVIDs in mice (n = 6). **(G)** Tissue Doppler imaging was employed to measure E/A to evaluate the diastolic function (n = 6). **(H-I)** Quantitative results of WGA and PSR staining in hearts (n = 6). All data are expressed as the mean ± S.D., and analyzed using one-way ANOVA followed by Tukey post hoc test. **P*< 0.05versus the matched group.

**
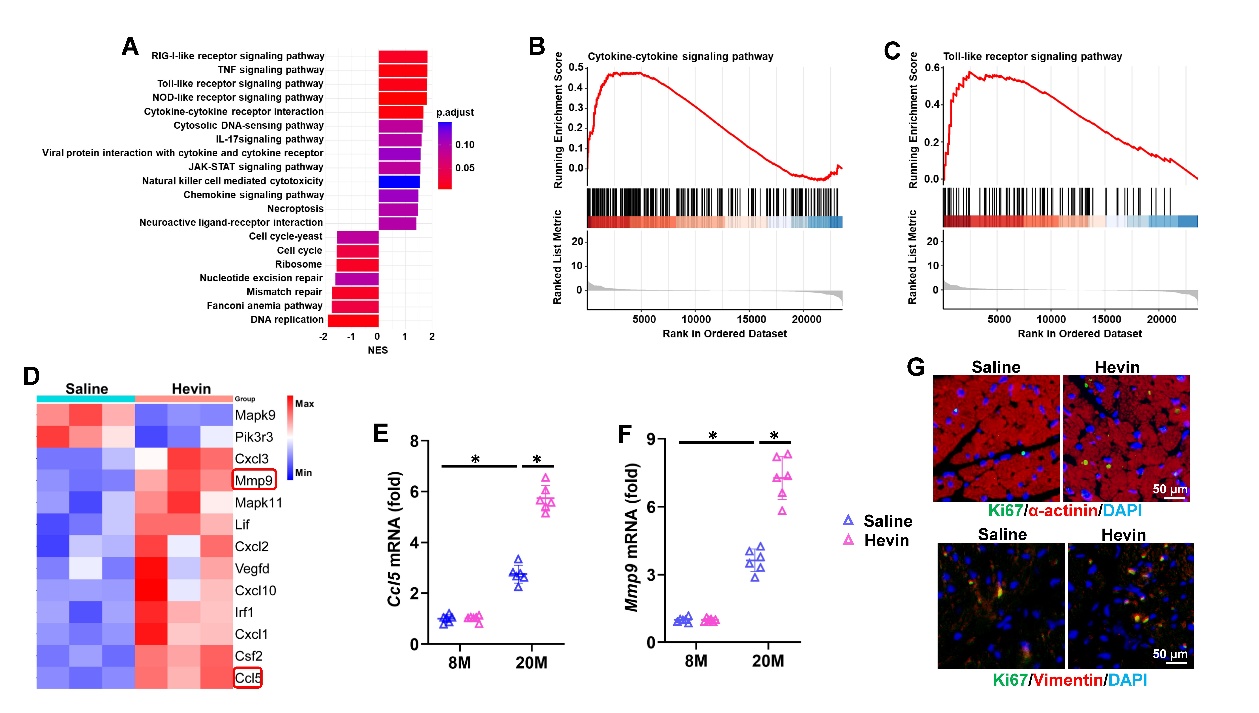
**

**Figure S8. Hevin administration stimulates CCL5 expression.**

**(A)** GSEA KEGG barplot of RNA-seq. **(B)** GSEA of cytokine-cytokine signaling pathway. **(C)** GSEA of toll-like receptor signaling pathway. **(D)** Heat maps showing the genes of TNF signaling pathway. **(E-F)** Relative *Ccl5* and *Mmp9* mRNA levels in hearts (n = 6). **(G)** Represent immunofluorescence image in aging hearts with or without Hevin administration (n = 6). All data are expressed as the mean ± S.D., and analyzed using one-way ANOVA followed by Tukey post hoc test. **P*< 0.05versus the matched group.

**
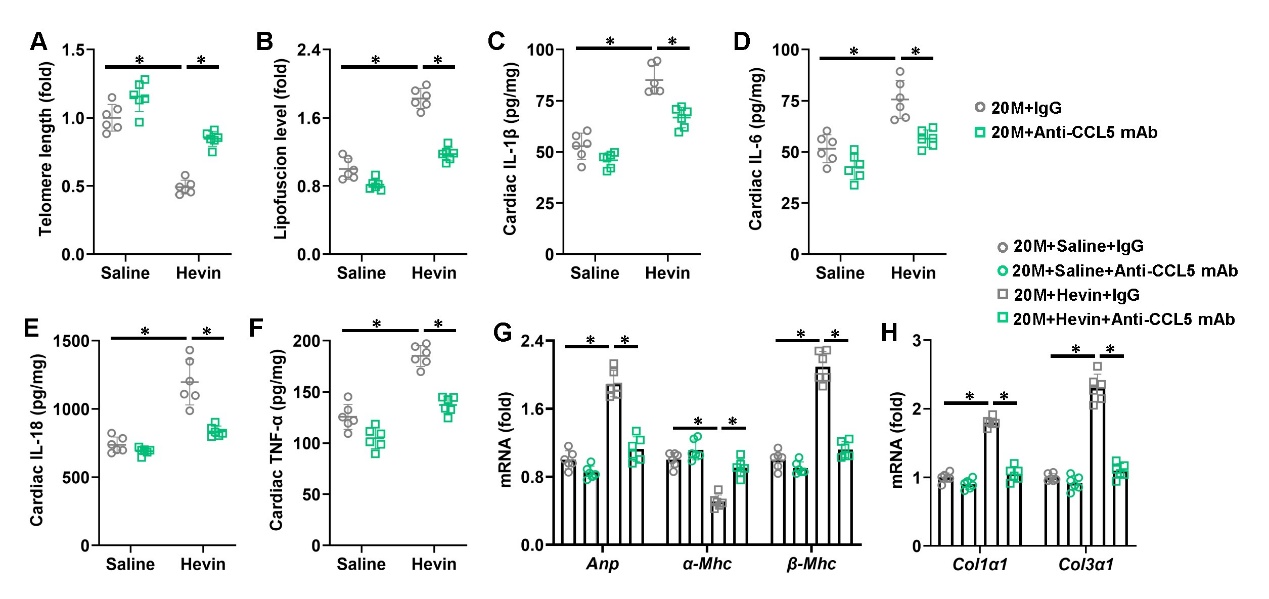
**

**Figure S9. Hevin exacerbates age-related cardiac inflammation and dysfunction by inducing CCL5.**

**(A)** Relative telomere length in hearts (n = 6). **(B)** Relative lipofuscin accumulation in hearts (n = 6). **(C-F)** The myocardial IL-1β, IL-18, IL-6 and TNF-α levels were determined by ELISA kits (n = 6). **(G)** Relative *Anp*, *α-Mhc* and *β-Mhc* mRNA levels in hearts (n = 6). **(H)** Relative *Col1α1* and *Col3α1* mRNA levels in hearts (n = 6). All data are expressed as the mean ± S.D., and analyzed using one-way ANOVA followed by Tukey post hoc test. **P*< 0.05versus the matched group.


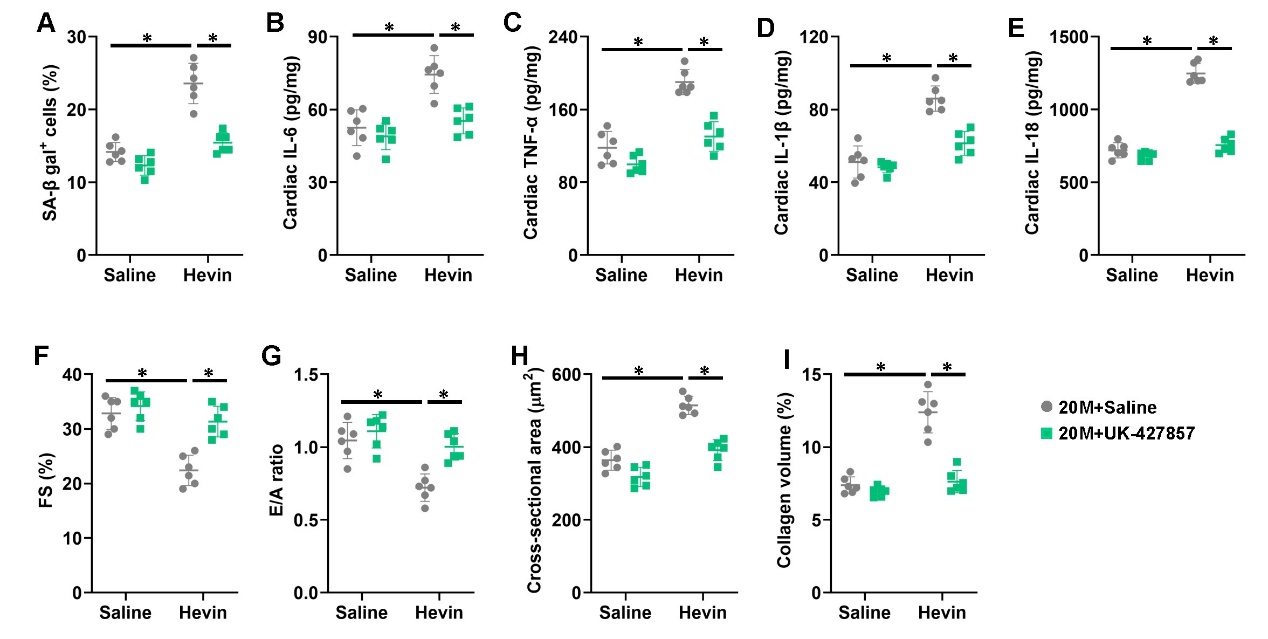


**Figure S10. Inhibition CCL5 with UK-427857** **ameliorates age-related cardiac inflammation and dysfunction.**

**(A)** Quantitative results of SA-β gal staining in hearts (n = 6). **(B-E)** The myocardial IL-1β, IL-18, IL-6 and TNF-α levels were determined by ELISA kits (n = 6). **(F)** Echocardiographic and hemodynamic parameters of cardiac function in mice, including FS in mice (n = 6). **(G)** Tissue Doppler imaging was employed to measure E/A to evaluate the diastolic function (n = 6). **(H-I)** Quantitative results of WGA and PSR staining in hearts (n = 6). All data are expressed as the mean ± S.D., and analyzed using one-way ANOVA followed by Tukey post hoc test. **P*< 0.05versus the matched group.

**
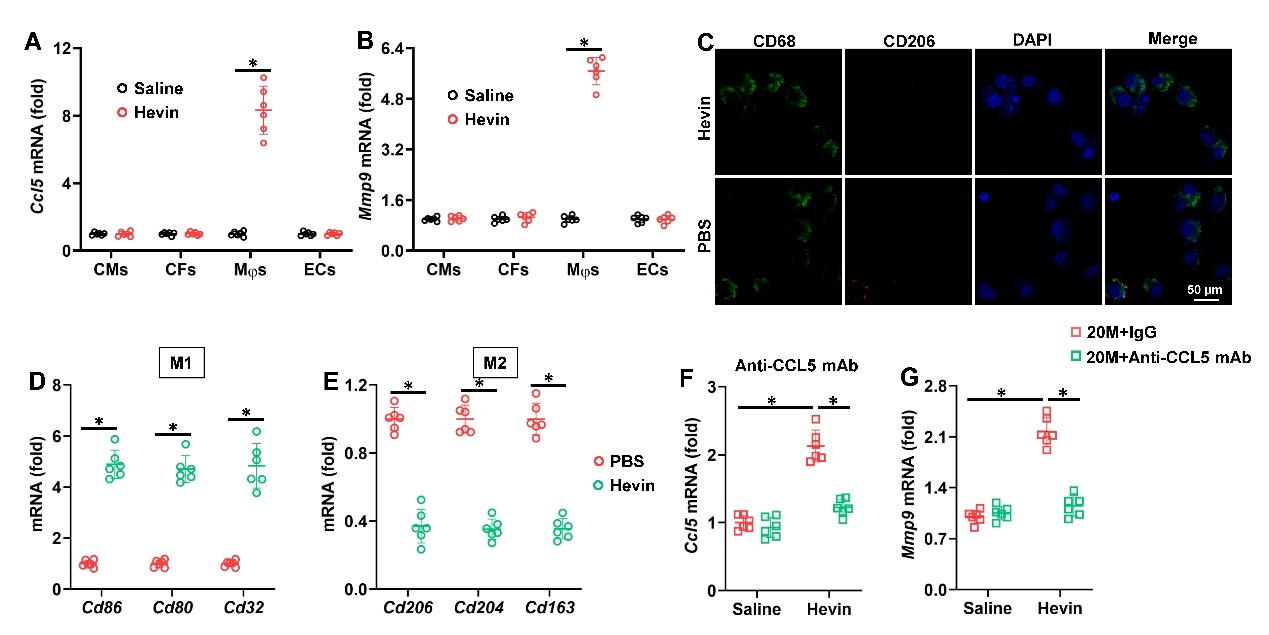
**

**Figure S11. CCL5 promotes macrophage polarization in the aging heart.**

**(A-B)** Relative *Ccl5* and *Mmp9* mRNA levels in different cells (n = 6). **(C)** Representative image of CD86 and CD68 staining in RAW264.7 (n = 6). **(D)** Relative *Cd86, Cd80* and *Cd32* mRNA levels in RAW264.7 (n = 6). **(E)** Relative *Cd206, Cd204* and *Cd163* mRNA levels in RAW264.7 (n = 6). **(F-G)** Relative *Ccl5* and *Mmp9* mRNA levels in hearts (n = 6). All data are expressed as the mean ± S.D., and analyzed using one-way ANOVA followed by Tukey post hoc test. **P*< 0.05versus the matched group.


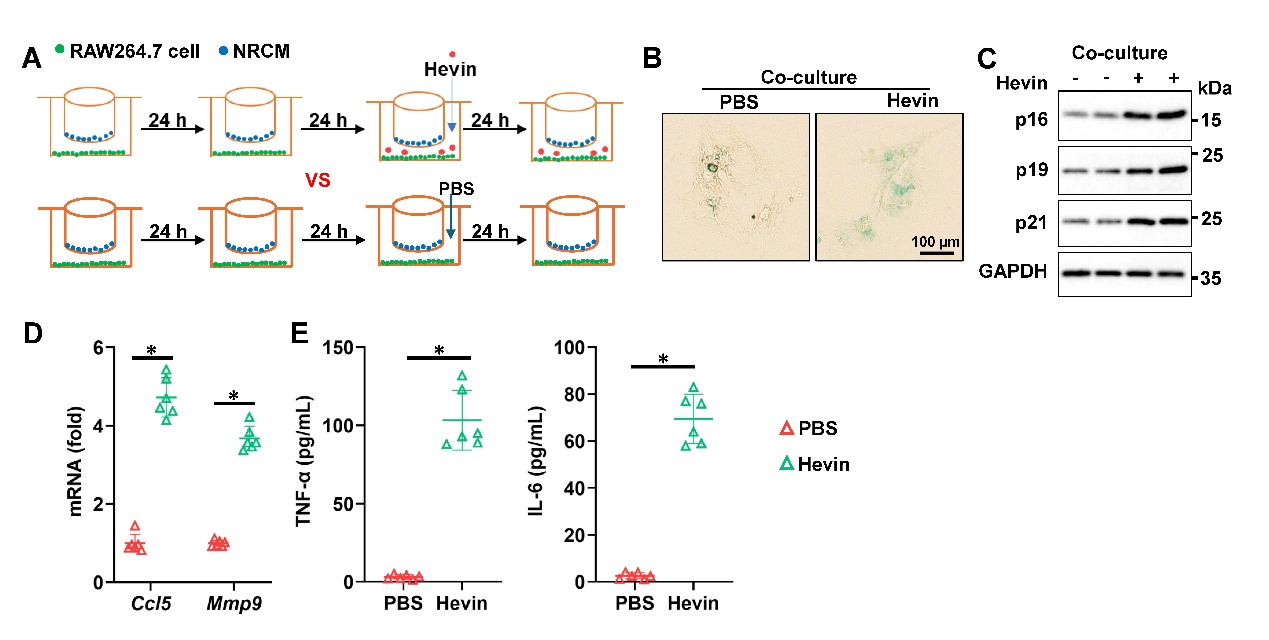


**Figure S12. Co-culture RAW264.7 cell and NRCM with Hevin induced NRCMs senescence.**

**(A)** Schematic protocol for co-culture RAW264.7 cell and NRCM in vitro. **(B)** Representative pictures of SA-β gal-stained cell (n = 6). **(C)** Western blot images of p16, p19, and p21 in NRCMs (n = 6). **(D)** Relative *Ccl5* and *Mmp9* mRNA levels in monocyte-derived Mφ (n = 6). **(E)** TNF-α and IL-6 levels were measured in monocyte-derived Mφ using commercial kit (n = 6). All data are expressed as the mean ± S.D., and analyzed using one-way ANOVA followed by Tukey post hoc test. **P*< 0.05versus the matched group.

**
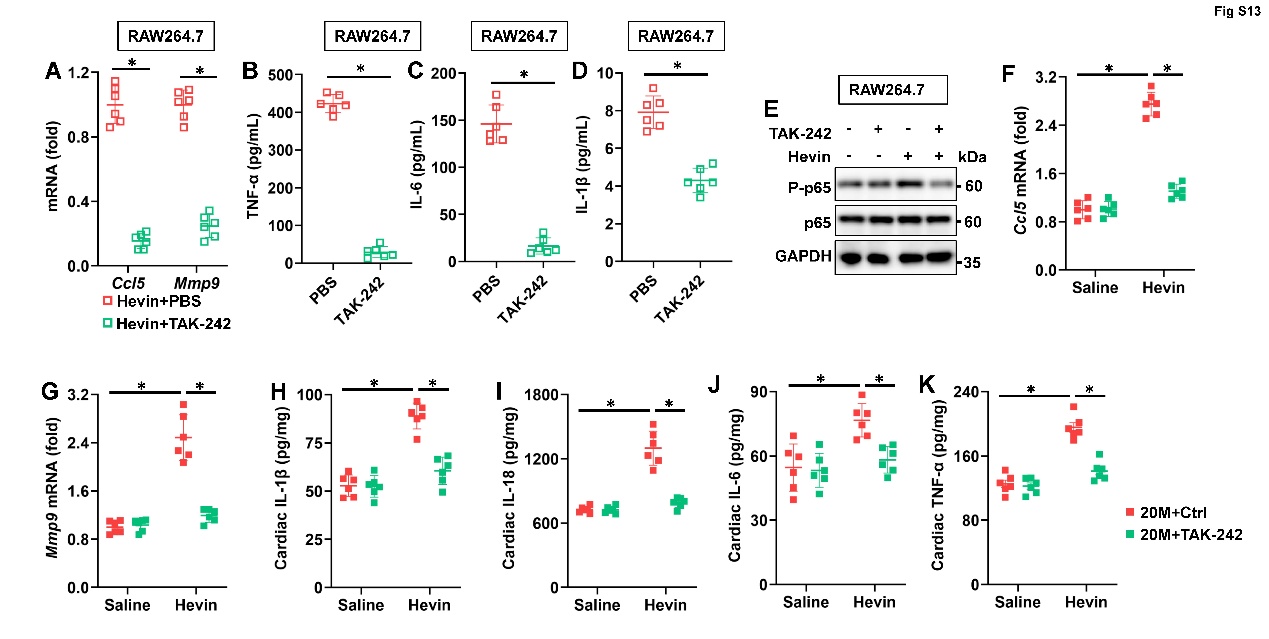
**

**Figure S13. Hevin regulates CCL5 expression through binding to TLR4 and activation of the p65.**

**(A)** Relative *Ccl5* and *Mmp9* mRNA levels in RAW264.7 cells (n = 6). **(B-D)** The IL-1β, IL-6 and TNF-α levels were determined by ELISA kits in RAW264.7 cells (n = 6). **(E)** Western blot images of p6 and P-p65 in hearts (n = 6). **(F-G)** Relative *Ccl5* and *Mmp9* mRNA levels in hearts (n = 6). **(H-K)** The myocardial IL-1β, IL-18, IL-6 and TNF-α levels were determined by ELISA kits (n = 6). All data are expressed as the mean ± S.D., and analyzed using one-way ANOVA followed by Tukey post hoc test. **P*< 0.05versus the matched group.

**
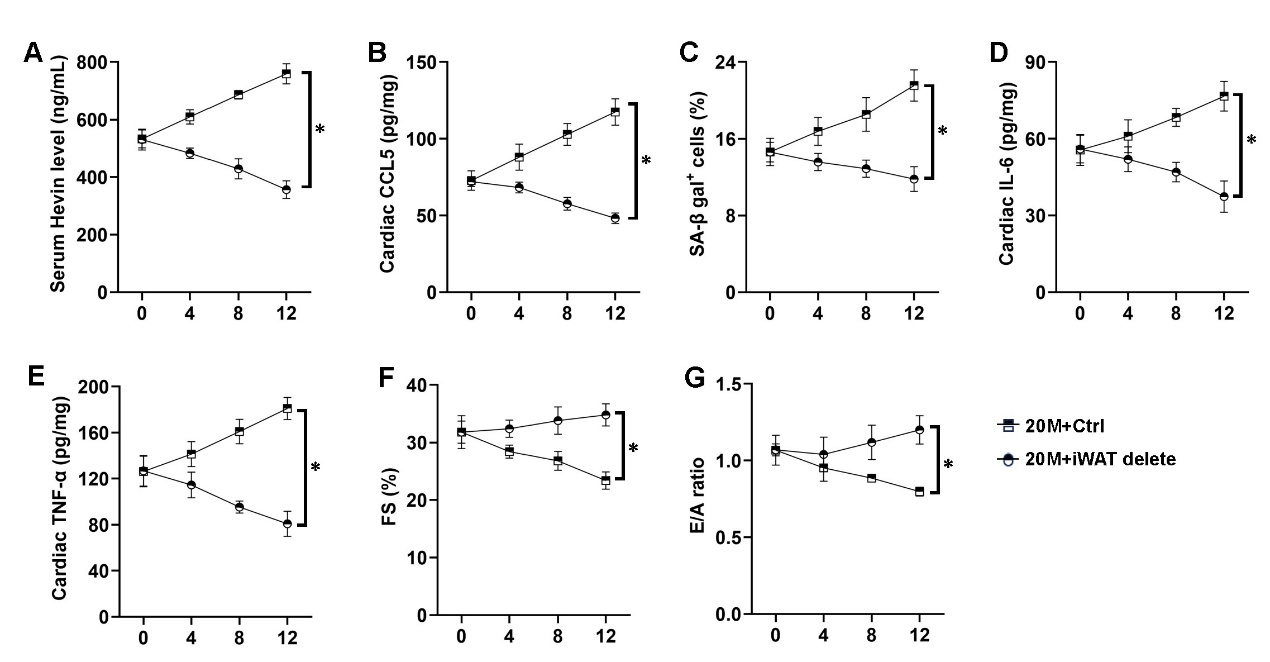
**

**Figure S14. Excision of iWAT improved the aging related cardiac dysfunction**

**(A)** Serum Hevin levels in mice (n = 6). **(B)** Cardiac CCL5 levels were measured in mice hearts using a commercial kit (n = 5). **(C)** Quantitative results of SA-β gal staining in hearts (n = 5). **(D-E)** The myocardial IL-6 and TNF-α levels were determined by ELISA kits (n = 5). **(F)** Echocardiographic and hemodynamic parameters of cardiac function in mice, including FS in mice (n = 5). **(G)** Tissue Doppler imaging was employed to measure E/A to evaluate the diastolic function (n = 5). All data are expressed as the mean ± S.D., and analyzed using one-way ANOVA followed by Tukey post hoc test. **P*< 0.05versus the matched group.
